# Artificial intelligence enabled parabolic response surface platform identifies ultra-rapid near-universal TB drug treatment regimens comprising approved drugs

**DOI:** 10.1371/journal.pone.0215607

**Published:** 2019-05-10

**Authors:** Daniel L. Clemens, Bai-Yu Lee, Aleidy Silva, Barbara Jane Dillon, Saša Masleša-Galić, Susana Nava, Xianting Ding, Chih-Ming Ho, Marcus A. Horwitz

**Affiliations:** 1 Division of Infectious Diseases, Department of Medicine, University of California, Los Angeles, California, United States of America; 2 Department of Mechanical and Aerospace Engineering, University of California, Los Angeles, California, United States of America; 3 Med-X Research Institute, School of Biomedical Engineering, Shianghai Jiao Tong University, Shanghai, China; 4 Department of Bioengineering, University of California, Los Angeles, California, United States of America; Institut de Pharmacologie et de Biologie Structurale, FRANCE

## Abstract

**Background:**

Shorter, more effective treatments for tuberculosis (TB) are urgently needed. While many TB drugs are available, identification of the best regimens is challenging because of the large number of possible drug-dose combinations. We have found consistently that responses of cells or whole animals to drug-dose stimulations fit a parabolic response surface (PRS), allowing us to identify and optimize the best drug combinations by testing only a small fraction of the total search space. Previously, we used PRS methodology to identify three regimens (PRS Regimens I–III) that in murine models are much more effective than the standard regimen used to treat TB. However, PRS Regimens I and II are unsuitable for treating drug-resistant TB and PRS Regimen III includes an experimental drug. Here, we use PRS methodology to identify from an expanded pool of drugs new highly effective near-universal drug regimens comprising only approved drugs.

**Methods and findings:**

We evaluated combinations of 15 different drugs in a human macrophage TB model and identified the most promising 4-drug combinations. We then tested 14 of these combinations in *Mycobacterium tuberculosis*-infected BALB/c mice and chose for PRS dose optimization and further study the two most potent regimens, designated PRS Regimens IV and V, consisting of clofazimine (CFZ), bedaquiline (BDQ), pyrazinamide (PZA), and either amoxicillin/clavulanate (AC) or delamanid (DLM), respectively. We then evaluated the efficacy in mice of optimized PRS Regimens IV and V, as well as a 3-drug regimen, PRS Regimen VI (CFZ, BDQ, and PZA), and compared their efficacy to PRS Regimen III (CFZ, BDQ, PZA, and SQ109), previously shown to reduce the time to achieve relapse-free cure in mice by 80% compared with the Standard Regimen (isoniazid, rifampicin, PZA, and ethambutol). Efficacy measurements included early bactericidal activity, time to lung sterilization, and time to relapse-free cure. PRS Regimens III–VI all rapidly sterilized the lungs and achieved relapse-free cure in 3 weeks (PRS Regimens III, V, and VI) or 5 weeks (PRS Regimen IV). In contrast, mice treated with the Standard Regimen still had high numbers of bacteria in their lungs after 6-weeks treatment and none achieved relapse-free cure in this time-period.

**Conclusions:**

We have identified three new regimens that rapidly sterilize the lungs of mice and dramatically shorten the time required to achieve relapse-free cure. All mouse drug doses in these regimens extrapolate to doses that are readily achievable in humans. Because PRS Regimens IV and V contain only one first line drug (PZA) and exclude fluoroquinolones and aminoglycosides, they should be effective against most TB cases that are multidrug resistant (MDR-TB) and many that are extensively drug-resistant (XDR-TB). Hence, these regimens have potential to shorten dramatically the time required for treatment of both drug-sensitive and drug-resistant TB. If clinical trials confirm that these regimens dramatically shorten the time required to achieve relapse-free cure in humans, then this radically shortened treatment has the potential to improve treatment compliance, decrease the emergence of drug resistance, and decrease the healthcare burden of treating both drug-sensitive and drug-resistant TB.

## Introduction

Tuberculosis (TB) is a health problem of global proportions. Almost one quarter of the world’s population is tuberculin-positive and potentially latently infected with *Mycobacterium tuberculosis* and these people have a 10% life-time risk of developing active TB [1]. In 2017, ten million people developed active TB and 1.3 million died of TB, making it the leading cause of death from a single infectious agent [1]. The current standard treatment for drug sensitive TB requires 6–9 months and is often complicated by problems with adherence, toxicity, and the development of antibiotic resistance. Treatment of drug-resistant TB is even more problematic, often requiring 20–26 months treatment with second and third line drugs [2] and is complicated by drug toxicities, treatment failure and non-completion [3].

Shorter and more effective treatments for TB are urgently needed to decrease the global burden of TB and to combat the emergence of drug resistance. While one important strategy is the development of new drugs, an equally important consideration is the identification of the most effective combinations of TB drugs, including both new and old TB drugs. A challenge in screening and identification of more effective multi-drug regimens is that the number of possible drug-dose combinations to be tested increases exponentially with the number of drugs. As we have described in detail previously [4], for N different drugs at M different dose levels, there are M^N^ different drug-dose combinations, such that for 15 different drugs at 5 different dose levels, there are 30.5 billion different drug-dose combinations. However, we have found in multiple studies that the drug-dose response surface is a smooth parabolic surface [4–6]. This has allowed us to develop an artificial intelligence enabled parabolic response surface (PRS) platform to model the drug-dose response surface and identify the most promising drug regimens by testing only a small portion of the total search space [4,5]. The PRS approach is a short-cut to identifying highly synergistic drug regimens, which otherwise would require testing billions of possible drug-dose combinations because both the drug and the drug dose impact the efficacy of a combination. The basic premise of the PRS approach, which has been evaluated in numerous biological systems, is that the efficacy of drugs at different doses is described by a smooth parabolic surface–in other words there are no abrupt changes in efficacy as dose is altered. Such a smooth parabolic surface is described by a second order algebraic equation. Therefore, to identify optimal drug-dose combinations, one does not need to test billions of possible drug-dose combinations but only to solve this algebraic equation by testing a relatively few drug-dose combinations over the surface in an iterative process. The PRS platform can save orders of magnitude effort, time and cost compared with a conventional search-all approach. Consequently, the PRS platform makes possible the optimization of both drug and dose in combinatorial therapy, especially *in vivo* treatment.

In a proof of principle, we used this strategy previously to identify from a set of 14 different drugs, several 4-drug combination regimens that were dramatically more effective in an *in vitro M*. *tuberculosis*–infected human macrophage model of TB than the current standard 4-drug regimen of isoniazid (INH), rifampicin (RIF), pyrazinamide (PZA), and ethambutol (EMB) [4]. We subsequently optimized two of these 4-drug regimens by combinatorial testing in a BALB/c mouse model of pulmonary TB and demonstrated that they dramatically reduced the time required to achieve relapse-free cure [5]. Whereas the Standard Regimen required 16 weeks to achieve relapse-free cure in our BALB/c mouse model of pulmonary TB, in the same experiment, the first regimen, designated PRS Regimen I and consisting of clofazimine (CFZ), prothionamide (PRO), PZA, and EMB, achieved relapse-free cure in 12 weeks, and the second regimen, designated PRS Regimen II and consisting of CFZ, EMB, PZA, and bedaquiline (BDQ), achieved relapse-free cure in only 4 weeks [5]. Our macrophage studies showed that EMB and the investigational TB drug, SQ109, were interchangeable. However, *M*. *tuberculosis* that is resistant to EMB remains sensitive to SQ109. We therefore tested the regimen CFZ, SQ109, PZA, BDQ (PRS Regimen III) and showed that it was as effective as PRS Regimen II and that both regimens achieved relapse-free cure in only 4 weeks in standard BALB/c mice (vs. 20 weeks for the Standard Regimen in the same experiment) as well as in the highly susceptible C3HeB/FeJ (Kramnik) mice [7].

In the current study, we expanded our macrophage screening assay to include the potent new TB drug delamanid (DLM), which is approved in Japan and has received conditional marketing approval by the European Medicines Agency for treatment of MDR-TB. Based on our expanded macrophage screening assays of combinations of 15 different drugs, we identified 14 four-drug regimens for further testing in the BALB/c mouse model of pulmonary TB. Based on this screening test, we selected the two most promising of these regimens for dose optimization using our PRS platform and subsequently for further testing of their efficacy in the murine TB model. These two regimens (designated PRS Regimens IV and V) had three drugs in common (CFZ, BDQ, and PZA) and differed only in their fourth drug, amoxicillin/clavulanate (AC) or DLM, respectively. In our drug-dose response optimization studies, we noted that in the case of both regimens, the optimized dose of this 4^th^ drug was relatively low, both with respect to its typical dosing in mouse TB treatment studies and the corresponding human equivalent dosing in current clinical practice, prompting us to test the three drug combination, consisting of CFZ, BDQ, and PZA (designated PRS Regimen VI), for efficacy in the murine TB model. We show here that all three of these regimens rapidly sterilize lung tissue and achieve relapse-free cure in the mouse BALB/c model of pulmonary TB in only 3 (PRS Regimens V and VI) or 5 (PRS Regimen IV) weeks. PRS Regimens V and VI were equivalent in efficacy to benchmark PRS Regimen III, previously shown to reduce the time to achieve relapse-free cure in mice by 80% compared with the Standard Regimen.

## Materials and methods

### TB drugs

Anti-TB drugs amoxicillin/clavulanate (AC 4:1 wt:wt ratio), clofazimine (CFZ), cycloserine (CYS), ethambutol (EMB), isoniazid (INH), linezolid (LZD), moxifloxacin (MXF), para-aminosalicyclic acid (PAS), prothionamide (PRO), pyrazinamide (PZA), and rifampicin (RIF), rifapentine (RPT), and streptomycin (STR) were purchased from Sigma-Aldrich. Bedaquiline (BDQ) was purchased from Asclepia (Belgium). PA-824 (PA824) was provided by the Global Alliance for TB Drug Development; SQ-109 (SQ109) was provided by Sequella, Inc; and delamanid (DLM) was provided by Otsuka Pharmaceutical Co. As described in detail previously [4], the various PRS experimental regimens and the Standard Regimen (INH, RIF, EMB, PZA) were mixed in sterile polypropylene 96-well deep well plates (Nunc) using Microlab STAR Line liquid handling workstation (Hamilton) operated by Venus One software.

### Preparation of *M*. *tuberculosis* for macrophage cell culture infection

We used the isopropyl β-D-1-thiogalactopyranoside (IPTG)-inducible ultraviolet-light optimized green fluorescent protein variant (GFPuv) derivative of the *M*. *tuberculosis* Erdman strain (Mtb-iGFP) [8] for our *in vitro* macrophage cell culture fluorescence-based inhibition assay as described previously [4]. A glycerol stock of the Mtb-iGFP strain was incubated on 7H11 agar plates containing hygromycin (50 μg/ml) and kanamycin (15 μg/ml) at 37°C, 5% CO_2_-95% air for 10 days. As described in detail previously [4], bacterial lawns were scraped from the agar plates into RPMI-1640 supplemented with 20 mM HEPES and the suspension was sonicated in a water bath sonicator (Astrason) for 8 periods of 15 seconds, with cooling of the suspension in an ice bath in between sonications to disperse aggregates. Residual aggregates were pelleted by centrifugation at 200 g for 10 min at 4°C and the bacterial suspension in the supernate was centrifuged again under the same conditions; this process was repeated for a total of five times. Optical density of the final suspension was measured with a GENESYS 10S UV-Vis spectrophotometer (Thermo Scientific) at 540 nm. The bacteria were opsonized with 10% human serum type AB in RPMI at 37°C for 10 min at a bacterial optical density of 0.1, diluted 40-fold to a bacterial optical density of 0.005 (7.5 x 10^6^ bacteria/ml) and 0.1 ml used to infect THP-1 macrophages.

### THP-1 macrophages

As described in detail previously [4], human THP-1 monocytic cells (American Type Culture Collection, TIB-202) were maintained in RPMI-1640 (Lonza) supplemented with 10% heat-inactivated fetal bovine serum (HI-FBS, Mediatech), 2 mM GlutaMAX (Life Technology), penicillin (100 IU) and streptomycin (100 μg/mL) at 37°C, 5% CO_2_−95% air atmosphere. For use in infection studies, the THP-1 cells were differentiated into macrophages in 96-well black glass bottom Matriplates (Brooks, Life Science Systems) with 100 nM phorbol 12-myristate 13-acetate (PMA; Sigma) in antibiotic-free RPMI with 10% HI-FBS in in RPMI containing 10% heat-inactivated fetal bovine serum (HI-FBS) at a density of 10^5^ THP-1 cells in 0.2 ml per well.

### High-throughput fluorescence based *M*. *tuberculosis*-macrophage infection assay

We employed a modification of our previously described high-throughput fluorescence based *M*. *tuberculosis*-macrophage infection assay [4]. Monolayers of PMA-differentiated THP-1 cells in black 96-well glass-bottom Matriplates were infected for 3 h with Mtb-iGFP at a ratio of 7.5:1 prior to incubating in medium with 1 mM IPTG and experimental TB drug combinations. Included as controls in each 96-well plate were: wells not infected with Mtb-iGFP (No Infection Control); wells to which the inducer IPTG was not added (No IPTG Control); and wells to which TB drugs were not added (No Drug Control). We assayed all conditions in triplicate using randomized well positions to control for location artifacts. We incubated the cultures for 4 days at 37°C, 5% CO_2_-95% air, fixed them for 1 h in 4% paraformaldehyde in Dulbecco’s Phosphate Buffered Saline (PBS), and stained the cell nuclei for 10 min with 1 μg/ml Hoechst 33342 in PBS containing 0.1% Tween 20. We washed the monolayers twice with PBS and imaged them with an ImageXpress (Molecular Devices) high throughput epifluorescence microscope using a 10x objective lens. We acquired three pairs of GFP and Hoechst epifluorescence images from non-overlapping regions of each well using fluorescein isothiocyanate (FITC) and 4′,6-diamidino-2-phenylindole (DAPI) filter cubes, respectively. We performed automated image analysis using the Granularity and Count Nuclei modules of MetaXpress (Molecular Devices) software to quantitate the integrated GFP fluorescence intensity and the number of macrophage nuclei, respectively, for each area imaged. We defined “inhibition” by the following equation:

$$Inhibition=1-\left(\frac{Integrated\;GFP\;Fluorescence\;Intensity\;per\;Nucleus\;of\;Treated\;Sample}{Integrated\;GFP\;Fluorescence\;Intensity\;per\;Nucleus\;of\;Untreated\;Sample}\right)$$ (Eq 1)

### PRS methodology applied to macrophage infection assay

In monotherapy, a higher dose generally gives higher efficacy. In combinatorial therapy, however, the optimal dose is usually much lower than the maximum tolerated dose due to drug-drug interactions. In other words, doses become an additional dimension in the search domain. As noted above, with 5 doses for a pool of 15 drugs, the search space has 5^15^ = 30.5 x 10^9^ drug-dose combinations, which is prohibitively large for the conventional search-all approach. Assisted by artificial intelligence analysis [9], the relationship between the regimen and inhibition of *M*. *tuberculosis* fluorescence in macrophage culture was found to be a parabolic shaped surface, which is governed by a second order algebraic equation (Eq 2) [4].

$$y=\beta_{0}+\beta_{1}x_{1}+\cdots +\;\beta_{n}x_{n}+\;\beta_{12}x_{1}x_{2}+\cdots +\beta_{mn}x_{m}x_{n}+\beta_{11}x_{1}^{2}+\cdots +\beta_{nn}x_{n}^{2}$$ (Eq 2)

Where *y* represents percentage of inhibition; *β*_*o*_ is the intercept term; *x*_*n*_ is the *n*^th^ drug dosage; *β*_*n*_ is the coefficient of the *n*^th^ drug; *β*_*mn*_ is the interaction coefficient between the *m*^th^ and *n*^th^ drugs; and *β*_*nn*_ is the quadratic coefficient for the *n*^th^ drug.

PRS screening for optimized TB drug regimens was performed in two steps. In the first step, we determined the drug-dose response curve for each individual drug in our macrophage model of *M*. *tuberculosis* infection to identify the concentration range appropriate for testing [4]. For searching 15 drugs, Eq 1 has 136 coefficients. We selected 136 drug-dose pairs and uniformly distributed these drug-dose pairs in the entire search space according to an orthogonal-array central composite design (OACD) for better description of the landscape of the PRS. The efficacy is experimentally determined for each drug-dose pair. With this data set, we were able to solve the 136 coefficients. Once the coefficients of Eq 1 were determined, the optimal doses were obtained. PRS methodology can be applied *in vitro* [4], *in vivo* preclinically [5,7] and in personalized therapy [6,9].

### Animals

Six to eight week old female BALB/c mice purchased from The Jackson Laboratory were housed 5 per cage in Innovive ventilated caging systems equipped with HEPA-filtered exhaust in our dedicated UCLA animal facility with HEPA-filtered air supply. We weighed the mice weekly and inspected them daily for adequacy of food, water, bedding and health conditions. Following aerosol infection, we monitored the mice daily for signs of illness (ruffled fur, decreased activity, hunched posture and/or tachypnea). Any mouse showing weight loss or signs of illness was weighed daily to insure adherence to a 10% weight loss limit. We promptly euthanized any mouse showing signs of illness and a weight loss of >10% of its initial weight. Euthanasia was achieved by hypercarbia using a gradual filling method with a flow rate of 20% volume displacement per min (i.e. a flow rate of 1.5 liters/min for a cage size of 7.5” x 11.75” x 5”). Mice were maintained under CO_2_ flow for at least one minute after respiratory arrest. Cervical dislocation was performed as a confirmatory euthanasia method prior to necropsy. All animal studies were approved by and conducted according to the procedures set forth by the UCLA Animal Research Committee (ARC # 1998–140). We used a total of 498 mice in the experiments described.

### Aerosol infection of BALB/c mice

*M*. *tuberculosis* strain Erdman (ATCC 35801) was harvested from infected outbred guinea pig spleens 3 weeks after aerosol infection. Spleens were homogenized in 7H9 medium and cultured on 7H11 agar without antibiotics. Bacteria were scraped from the plates with wire loops and suspended in 7H9 media containing 10% glycerol, sonicated in a water bath to disperse aggregates, and allowed to settle for 1 h. The supernate above the settled pellet was aspirated and stored frozen in 1 ml aliquots at -80°C. The titer of the aliquots was determined by thawing, plating serial dilutions on 7H11 agar, and enumerating colony forming units (CFU). Prior to an infection experiment, an aliquot was thawed, diluted to a concentration of 1.875 x 10^6^ CFU/ml in 20 ml of PBS, and used to infect BALB/c mice using a Collison 6-jet nebulizer fixed to a custom-designed Plexiglas aerosolization chamber contained within a Class II A2 biosafety cabinet. The 30 minute exposure used in these experiments delivered ~250 live bacteria to the lungs of each mouse. Two mice from each aerosolization experiment were euthanized one day after aerosolization to determine the number of organisms delivered to the lung. At the end of the pre-treatment period for each experiment (Day 14), an additional three mice were euthanized to determine *M*. *tuberculosis* CFU in the lung at the start of treatment.

### Antibiotic treatment

Treatment was begun two weeks (Day 14) after aerosol infection. Antibiotics were given daily 7 days/week by oral gavage (EBA_14_ study) or 5 days per week (Monday–Friday) for all other efficacy and relapse studies. For treatment with the Standard Regimen, RIF was given first followed at least one hour later by the combination of INH, EMB and PZA. For regimens containing RPT, the RPT was administered one hour prior to the other combined antibiotics. For regimens containing CFZ, the CFZ was suspended in 0.15% agarose and administered separately one hour after the other combined antibiotics (except for RPT). For regimens that included both RPT and CFZ, the RPT was given first, followed one hour later by the non-CFZ antibiotics, followed one hour later by the CFZ. RIF was administered in water; RPT was suspended in 5% gum arabic; and all other drugs were prepared as a suspension in 0.15% agarose. Sham-treated mice were given water and then 0.15% agarose suspension by oral gavage. Drugs administered and abbreviations used in this study are shown in Table 1.

**Table 1:** Abbreviation of TB drugs tested in the study.

Abbreviation	Drug
AC	Amoxicillin-Clavulanate
BDQ	Bedaquiline
CFZ	Clofazimine
CYS	Cycloserine
DLM	Delamanid, OPC-67683
EMB	Ethambutol
INH	Isoniazid
LZD	Linezolid
MXF	Moxifloxacin
PA824	PA-824
PAS	Para-aminosalicylic acid
PRO	Prothionamide
PZA	Pyrazinamide
RIF	Rifampicin
RPT	Rifapentine
SQ109	SQ-109
SR	Standard Regimen (INH, RIF, EMB, PZA)
PRS Regimen II	CFZ, BDQ, PZA, EMB
PRS Regimen III	CFZ, BDQ, PZA, SQ109
PRS Regimen IV	CFZ, BDQ, PZA, AC
PRS Regimen V	CFZ, BDQ, PZA, DLM
PRS Regimen VI	CFZ, BDQ, PZA

## Results

### Identification of drug combinations by screening in macrophage cell culture

We used our previously described PRS (FSC.II) method [4] to identify, from an initial set of 15 different TB drugs, the 3- and 4-drug combination regimens that are most effective in suppressing growth of *M*. *tuberculosis* in macrophages. The assay uses a high-throughput macrophage cell culture model of TB using *M*. *tuberculosis* that expresses IPTG-inducible green fluorescent protein (Mtb-iGFP). We selected and acquired 15 TB drugs for testing (Table 1), including the first-line drugs INH, RIF, PZA, EMB, second-line drugs MXF, PAS, PRO, CYS, and BDQ, third-line drugs AC, CFZ, LZD, DLM, and the investigational drugs PA824 and SQ109.

#### Phase 1: Establishment of dose response curve for each drug

As described in detail previously [4], we utilized a high-throughput fluorescence-based *M*. *tuberculosis*-macrophage infection assay to quantify the efficacy of TB drug combinations in inhibiting Mtb-iGFP in human macrophages in cell culture. This assay utilized a robotic fluorescence microscope for high-content image acquisition and automated image analysis to quantitate the inhibitory effect on *M*. *tuberculosis* of single and combinatorial anti-TB drugs. As a control, we studied the combination comprising the 1980s Standard Regimen (INH, RIF, EMB and PZA) [10]. The first phase of the PRS methodology determines the dose-response curve for each individual drug in the *M*. *tuberculosis*-macrophage infection system by serially diluting single drugs in culture medium containing the inducer IPTG, adding the solutions to Mtb-iGFP infected monolayers of THP-1 macrophages, and after 4 days of incubation, measuring the inhibition of bacterial growth by automated high-content image acquisition and analysis of the infected monolayers. We observed a direct relationship between drug concentration and degree of inhibition for all 15 drugs used in this study (S1 Fig) [4].

#### Phase 2: Screening test and iterations

After establishing the individual drug dose-response curves, we tested combinations of the 15 different drugs at dose levels that provide either 10% inhibition or 0% inhibition (i.e. no drug) (S1 Table). The drug combinations tested in this and subsequent screenings are selected using an Orthogonal Array design (S2–S7 Tables). This initial screening allowed us to fit a linear model to the data and to eliminate 4 of the 15 drugs (CYS, INH, LZD, and MXF) that showed antagonistic interactions and did not contribute to the combinatorial efficacy (Table 2). It is noteworthy that our previous *in vitro* macrophage screening study also rapidly eliminated these four drugs [4]. The second round of screening examined the remaining 11 drugs at three different dose levels (10%, 5%, or 0% inhibition) and eliminated two additional drugs that contributed little to combinatorial efficacy: PAS and PRO, leaving 9 drugs for study in the third round. The third round studied 9 drugs at three different dose levels (15%, 7.5%, and 0%) and led to elimination of EMB, leaving 8 drugs. The fourth round studied the remaining 8 drugs (AC, BDQ, CFZ, DLM, PA824, PZA, RIF, and SQ109) at 5 dose levels (20%, 15%, 10%, 5%, and 0%). A 5^th^ and 6^th^ round of testing was then conducted with 6 or 7 of these drugs (omitting PZA and/or PA824, respectively) to refine the response surface for these remaining drugs.

**Table 2:** Screening of combinatorial drugs in macrophage model of *M*. *tuberculosis* infection.

	Screening test	Iteration #1	Iteration #2	Iteration #3A	Iteration #3B	Iteration #3C
Effect levels^a^	2	3	3	5	5	3
Test runs^b^	150	102	155	75	75	82
Drugs^c^	AC	AC	AC	AC	AC	AC
	BDQ	BDQ	BDQ	BDQ	BDQ	BDQ
	CFZ	CFZ	CFZ	CFZ	CFZ	CFZ
	DLM	DLM	DLM	DLM	DLM	DLM
	RIF	RIF	RIF	RIF	RIF	RIF
	SQ109	SQ109	SQ109	SQ109	SQ109	SQ109
	PA824	PA824	PA824	PA824		PA824
	PZA	PZA	PZA	PZA		
	EMB	EMB	EMB			
	PAS	PAS				
	PRS	PRO				
	CYS					
	INH					
	LZD					
	MXF					

^a^Screening test was conducted at individual drug concentration that yielded 0% or 10% of the maximal inhibition to the IPTG-induced green fluorescence signal. Iteration #1 was conducted at individual drug concentration that gave 0% or 10% of the maximal inhibition level and one-half of the concentration that gave 10% of the maximal inhibition level. Iteration #2 was done at 0% or 15% effect levels and one-half of the concentration that gave 15% of the maximal inhibition level. Iterations #3A and 3B were conducted at 0% or 20% effect level and two-thirds, one-half or one-fourth of the concentration that gave 20% of the maximal inhibition level. Iteration #3C was conducted at 0% or 15% effect levels and one-half of the concentration that gave 15% of the maximal inhibition level.

^b^Number of combinatorial drug test runs in each experiment.

^c^List of drugs tested at screening and each stage of iterations.

**Fig 1:**
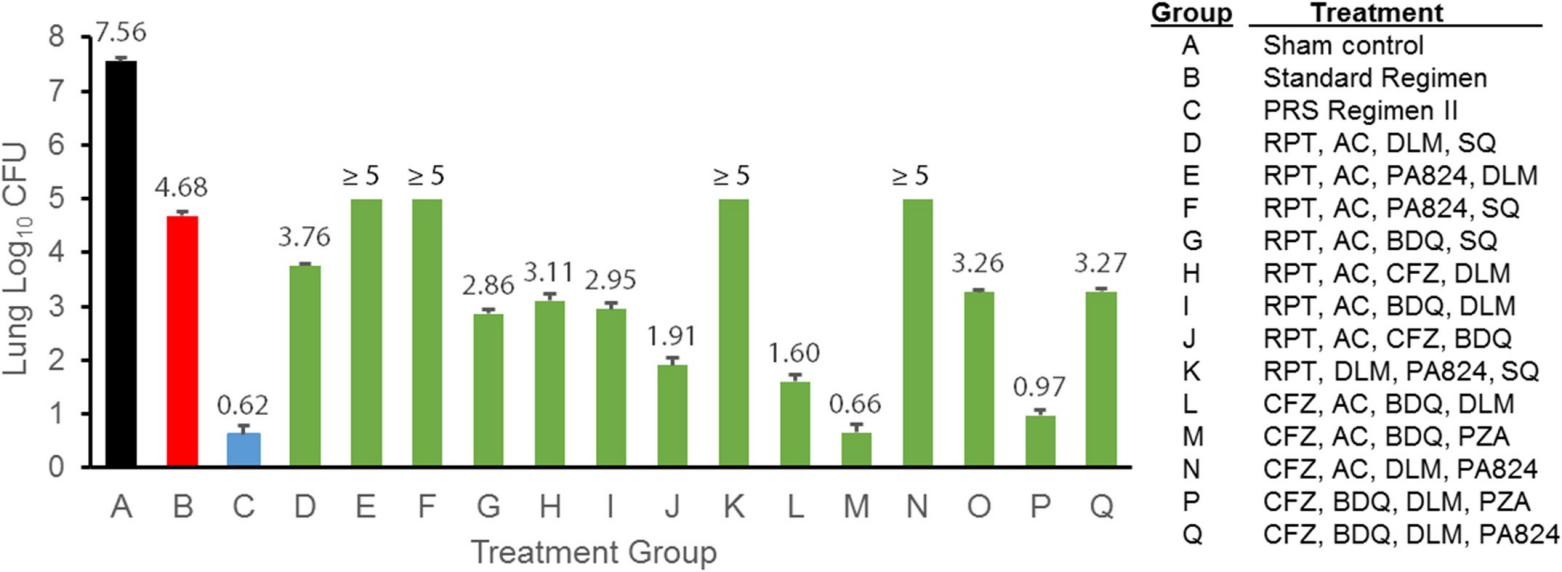
*In vivo* short-term efficacy screen of experimental regimens identified in macrophage studies using the PRS platform. *M*. *tuberculosis* infected mice were sham treated (Treatment Group A) or treated with the Standard Regimen (Treatment Group B), PRS Regimen II (Treatment Group C) or one of the top 4-drug combinations (Treatment Groups D-Q) identified from macrophage screening using the PRS platform starting from a pool of 15 TB drugs 5 days per week for 3 weeks. Three days after the last treatment, mice were euthanized to determine bacterial number in the lung. Standard Regimen is comprised of INH, RIF, EMB and PZA at 25, 10, 100 and 150 mg/kg, respectively. PRS Regimen II is comprised of CFZ, BDQ, PZA and EMB at 25, 30, 450 and 100 mg/kg, respectively. Drug doses used in the top 4-drug experimental regimens are as follows: 200–50 mg/kg for AC, 30 mg/kg for BDQ, 25 mg/kg for CFZ, 2.5 mg/kg for DLM, 100 mg/kg for PA824, 450 mg/kg for PZA, 10 mg/kg for RPT and 25 mg/kg for SQ109.

Based upon the results of these *in vitro* macrophage screening tests, we identified fourteen experimental regimens (labeled regimens D-Q in Fig 1) for further testing. All fourteen regimens are near universal regimens with at least three drugs in the 4-drug combination being either new drugs (i.e. PA824, SQ109, BDQ and DLM) or second-line drugs (i.e. AC, CFZ) that would retain efficacy in many patients with MDR- or XDR-TB.

### Comparison of 14 regimens in short-term efficacy study in mouse model of TB

We evaluated the treatment efficacy of these 14 new near universal regimens in a 3-week short-term screening study in a BALB/c model of pulmonary TB. We substituted rifapentine (RPT) for RIF in the regimens because it has the same mechanism of action and its longer half-life (10–15 h vs. 2–5 h, respectively) may provide greater efficacy in mouse models of TB [11,12]. For this study, we chose the following drug doses that have been found to be effective and well tolerated in previous long-term mouse TB studies: CFZ (25 mg/kg) [5,13,14], PA824 (100 mg/kg) [15–17], BDQ (30 mg/kg) [5], PZA (450 mg/kg) [5,18], RPT (10 mg/kg) [19,20], and SQ109 (25 mg/kg) [21]. In the case of DLM, while pharmacokinetic studies have used doses of up to 40 mg/kg [22], DLM has shown potent efficacy at 2.5 mg/kg [22,23] and 1 mg/kg [24] in long-term treatment studies in mouse TB models. We chose the dose of 2.5 mg/kg to be consistent with dosing in the previous TB treatment efficacy studies [22,23].

Clavulanate is a β-lactamase inhibitor that irreversibly inactivates mycobacterial β-lactamase [25], allowing amoxicillin/clavulanate (AC) to show activity against MDR-TB *in vitro* [26]. AC has been used clinically and showed an EBA_0-2_ of 0.34 log_10_ CFU when dosed at 1000/250 mg three times daily in previously untreated TB patients [27], though its EBA_0-2_ was no different from no drug when dosed once daily as a 3000/750 mg dose [28]. Amoxicillin/clavulanate has been used in combination therapy in patients with MDR-TB [29], and is considered a “group 5 drug” for use against MDR-TB and XDR-TB by the WHO. Treatment of *M*. *tuberculosis* infected BALB/c mice with a once-daily dose of 200 mg/kg amoxicillin and 50 mg/kg clavulanate gave a 2-log-unit reduction in the lung CFU in mice infected with an *M*. *tuberculosis* transpeptidase (LPD2) mutant, but no activity at that dose against the wild-type strain [30] or when AC was given 200/50 mg/kg BID in a BALB/c TB mouse model [31]. While studies using AC as a single agent in mice have shown poor activity [30,31], *in vitro* studies have shown synergistic interactions of AC with INH, RIF, and EMB [32,33], suggesting that drug combination studies may demonstrate efficacy due to synergy not seen with monotherapy. We used the dose of 200 mg/kg amoxicillin and 50 mg/kg clavulanate as done in the previous mouse TB treatment studies [30,31]. Typical doses of AC in other mouse infection models use ~100 mg/kg [34]. Based upon our experience with other TB drug regimens, using well-tolerated high doses in drug combination regimens yields fairly good, though not necessarily optimal inhibition of *M*. *tuberculosis in vivo*.

Ten out of the fourteen regimens were better than the Standard Regimen in reducing the lung burden of *M*. *tuberculosis* (Fig 1 and S8 Table). Among those, experimental regimens M and P were the two best regimens, which we designated as PRS Regimen IV (CFZ/BDQ/PZA/AC) and PRS Regimen V (CFZ/BDQ/PZA/DLM), respectively. These two regimens were markedly more effective than the other regimens and were of comparable efficacy to our previously published PRS Regimen II (group C in Fig 1). Intriguingly, these two regimens have three drugs (CFZ, PZA, BDQ) in common with our previously studied PRS Regimens II and III, and differ only in the fourth drug (EMB for PRS II, SQ109 for PRS III, AC for PRS IV, and DLM for PRS V).

In general, treatment groups that included either PA824 or RPT (or both) were less effective in reducing bacterial burden in the lungs than treatment groups that replaced these drugs with other drugs. For example, groups E, H, and I shared three drugs (RPT, AC, and DLM) and differ only in that PA824 of group E is replaced by CFZ in group H and by BDQ in group I. Both groups H and I were significantly more effective than group E (*p* < 0.0001, One-way ANOVA with Tukey’s correction for multiple comparisons), suggesting that PA824 is less synergistic or more antagonistic with the other three drugs than CFZ or BDQ.

Of the 16 regimens tested (14 new regimens, the Standard Regimen and PRS Regimen III), none of the eight RPT containing regimens were among the top four drug regimens. It is noteworthy that treatment group J in Fig 1 (RPT, AC, CFZ, BDQ) is the same as regimen M (PZA, AC, CFZ, BDQ = PRS Regimen IV) except that RPT of regimen J is replaced by PZA in regimen M. Regimen M is significantly more effective than regimen J (*p* < 0.0001, One-way ANOVA with Tukey’s correction for multiple comparisons), suggesting that PZA has better synergy or less antagonism with the other three drugs than RPT. In humans, both RIF and RPT have many adverse drug interactions related to their potent induction of drug metabolizing enzymes and are predicted to reduce steady-state plasma concentrations of BDQ by 79% and 75%, respectively [35]. However, in the mouse, RIF dosing does not induce BDQ metabolism [20], indicating that other factors are responsible for the superior sterilizing activity of PRS Regimen IV (Regimen M in Fig 1) vs. Regimen J.

The nitro-imidazole compounds DLM and PA824 are analogs and both inhibit mycolic acid synthesis and also may act as respiratory poisons by release of nitric oxide during their metabolism [22,36,37]. These compounds kill both aerobic, replicating *M*. *tuberculosis* and hypoxic, non-replicating *M*. *tuberculosis* and the respiratory poisoning mechanism is thought to underlie their activity against hypoxic, non-replicating *M*. *tuberculosis* [36,37]. The 4 treatment groups that combine these two drugs (i.e. groups E, K, N, and Q) were among the worst performing groups, and in these groups, replacing the PA824 component with one of the other drugs was consistently much more effective than the corresponding PA824 + DLM combination. For example, regimen K (RIF, PA824, SQ, DLM) was markedly less effective than regimen D (RIF, AC, SQ, DLM), though as individual drugs, PA824 is considerably more potent than AC, which has little or no activity as monotherapy in mice [30,31]. Likewise, regimens P and Q share CFZ, BDQ, and DLM, but PA824 of regimen Q is replaced by PZA in regimen P, making regimen P (PRS Regimen V) significantly more effective (*p* < 0.0001, One-way ANOVA with Tukey’s correction for multiple comparisons). We did not detect antagonism between PA824 and DLM in our *in vitro* macrophage screening assay; however, the poor performance of this combination in the mouse TB model suggests that they may have antagonistic interactions *in vivo*. Two antibiotics of the same class are occasionally used in combination clinically, e.g. double-beta-lactams for some infections, but in general it is preferable to combine different classes of antibiotics that target different pathways or at least different stages of the same pathway. Our data suggest that PA824 and DLM should not be used in combination.

### Optimization of drug dosing for PRS Regimens IV and V

**Fig 2:**
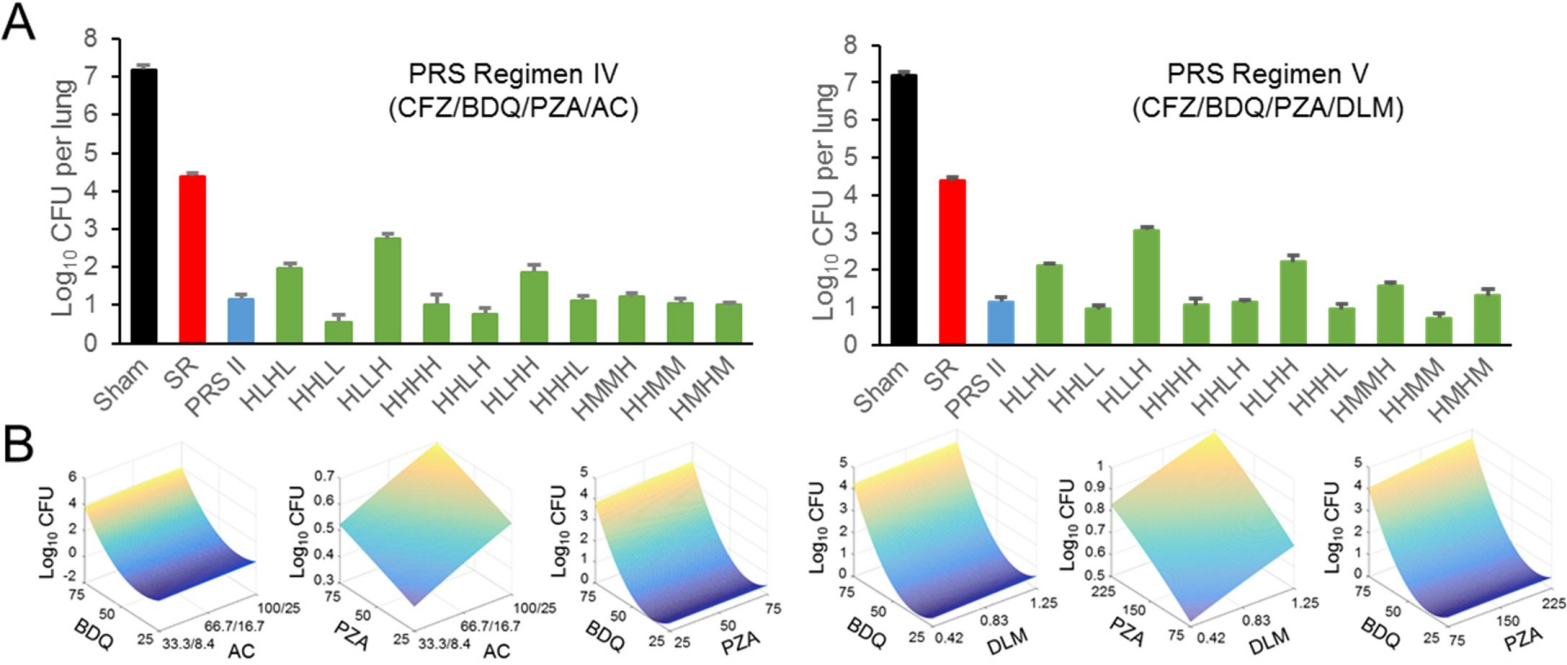
Optimization of PRS Regimens IV and V. (A) Lung burdens of *M*. *tuberculosis* in 10 groups of mice were determined after treatment 5 days per week for 3 weeks with PRS Regimen IV (top left) or PRS Regimen V (top right); the individual drugs were administered in high dose (H), middle dose (M) or low dose (L) as indicated below the horizontal axis. Sham-treated mice or mice treated with either the Standard Regimen (SR) or dose-optimized PRS Regimen II (PRS II) served as controls. The Standard Regimen comprised INH, RIF, EMB and PZA at 25, 10, 100 and 150 mg/kg, respectively. PRS Regimen II comprised CFZ, BDQ, PZA and EMB at 25, 30, 450 and 100 mg/kg, respectively. For PRS Regimen IV, the high doses for CFZ, BDQ, PZA and AC were 25, 50, 450 and 600–150 mg/kg, respectively. For PRS Regimen V, the high doses for CFZ, BDQ, PZA and DLM were 25, 50, 450 and 7.5 mg/kg, respectively. The middle dose of each drug was 1/3^rd^ that of the high dose and the low dose was 1/3^rd^ that of the middle dose. (B) Drug-dose efficacy response surface shown as drug-drug doses on the x- and z-axes and the projected lung log_10_ CFU on the y-axis.

We conducted short-term efficacy studies in our BALB/c mouse model of TB to map the phenotypic response surface for PRS Regimens IV and V (Fig 2). We treated ten groups of mice 5 days per week for three weeks with PRS Regimen IV or PRS Regimen V with the CFZ dose held constant at 25 mg/kg and the other three drugs (BDQ, PZA, and either AC or DLM, respectively) given at permutations of high, middle (1/3^rd^ of the high dose) or low (1/9^th^ of the high dose) doses as indicated in S9 Table. As controls, one group of mice received sham treatment and two other groups of mice were treated with the Standard Regimen or PRS Regimen II, respectively. Because these experiments were conducted concurrently, these three control groups were shared between the two experiments. For both PRS Regimens IV and V, all 10 of the experimental groups were more effective than the Standard Regimen in reducing CFU burden (Fig 2A and S9 Table). We fit algebraic quadratic equations to the phenotypic dose-response surface (Fig 2B) and used these equations to determine the optimum drug doses for each of these regimens. The phenotypic response surfaces indicated that optimal doses for PRS Regimen IV drugs BDQ, PZA, and AC are 37, 50, and 66.7/16.7 (amoxicillin/clavulanate) mg/kg, respectively; and the optimal doses for PRS Regimen V drugs BDQ, PZA, and DLM are 40, 185, and 0.83 mg/kg, respectively. For PRS Regimen IV, the optimized dose of AC was found to be the lowest dose tested (66.7/16.7 mg/kg) and for PRS regimen V, the optimized dose of DLM was also the same as the lowest dose tested (0.83 mg/kg). Therefore, to assess the contribution of the fourth drug in these regimens, we included an additional regimen in our subsequent testing, PRS Regimen VI, comprising only the 3 drugs in common between these two regimens (CFZ, BDQ, and PZA) at the doses used in PRS Regimen V.

### Determination of EBA_14_ of PRS Regimens IV, V, and VI

We determined the 14-day early bactericidal activity of our three regimens in our BALB/c mouse model of TB. Mice were infected by aerosol challenge with *M*. *tuberculosis* and treated by daily gavage (7 days per week for 14 days) with the Standard Regimen, PRS Regimens IV, V, or VI, or with vehicle (water and agarose suspension). Three days after their last gavage treatment, the mice were euthanized; the bacterial burden in their lungs was determined by plating for CFU and the EBA_14_ was calculated (Table 3). PRS regimens IV, V, and VI had similar log_10_ CFU EBA_14_ values (0.37, 0.36, and 0.36, respectively) and all showed significantly greater early bactericidal activity than the Standard Regimen (0.14, *p* < 0.0001, One-way ANOVA with Tukey’s correction for multiple comparisons).

**Table 3:** EBA_14_ study.

Treatment group^a^	Log_10_ CFU/lung		EBA14^b^
	Day 0	Day 14	
Sham control	6.75		
Standard Regimen		4.85	0.14
PRS Regimen IV		1.59	0.37
PRS Regimen V		1.72	0.36
PRS Regimen VI		1.67	0.36

^a^tandard Regimen is comprised of INH, RIF, EMB and PZA at 25, 10, 100 and 150 mg/kg, respectively. PRS Regimen IV is comprised of CFZ, BDQ, PZA and AC at 25, 37, 50 and 66.7–16.7 mg/kg, respectively. PRS Regimen V is comprised of CFZ, BDQ, PZA and DLM at 25, 40, 185 and 0.83 mg/kg, respectively. PRS Regimen VI is comprised of CFZ, BDQ and PZA at 25, 40 and 0.83 mg/kg, respectively.

^b^Average log_10_ CFU of *M*. *tuberculosis* reduction per day in the lungs of mice (n = 5 per group) during the first 14 days of treatment.

### Treatment efficacy of PRS Regimens IV, V, and VI

**Fig 3:**
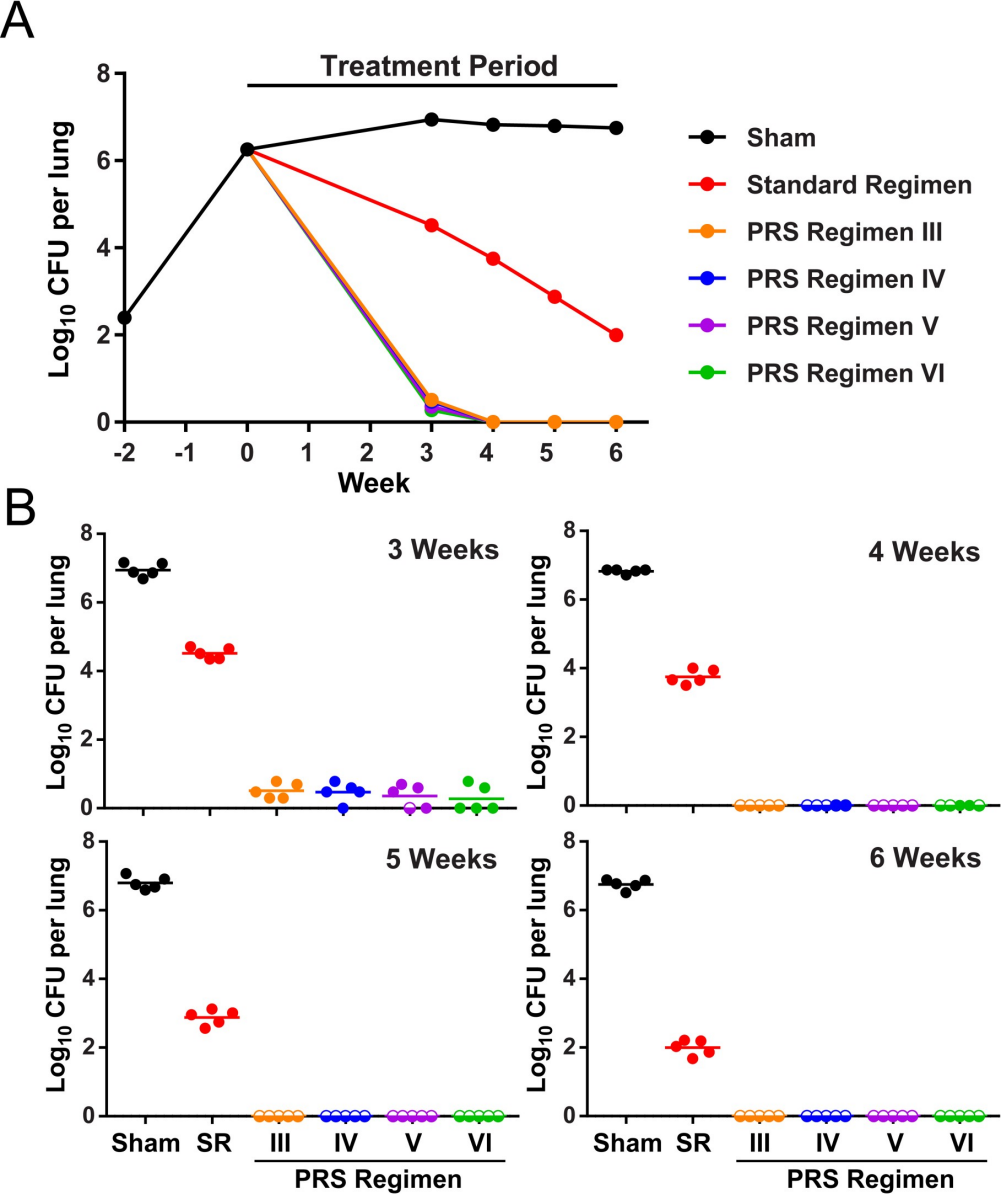
Treatment efficacy of PRS Regimens in BALB/c mice. (A) Time course of the bacterial burden in the lung over the infection and treatment period. (B) Lung burden of *M*. *tuberculosis* after treatment 5 days per week for 3, 4, 5, and 6 weeks in sham-treated mice or mice treated with the Standard Regimen (SR) or one of the PRS Regimens (III–VI). Mice with zero CFU in the lungs are plotted as log 0 CFU on the scale and indicated by a semi-open circle symbol.

We compared the treatment efficacy of PRS Regimens III, IV, V, and VI to the Standard Regimen and the sham control. Mice were infected by aerosol as described above, achieving 6.25 log CFU in the lungs after two weeks, whereupon treatment was initiated (Week 0) by oral gavage 5 days per week for 3, 4, 5, or 6 weeks (S10 Table). The mice were euthanized 3 days after their last gavage treatment and bacterial burden in the lungs was determined by plating for CFU. All four PRS regimens had similar efficacy curves (Fig 3A), achieving single digit numbers of CFU in the lungs after only 3 weeks of treatment and having no detectable CFU in the lungs or only a single CFU at weeks 4, 5, and 6 (Fig 3B). There was no significant difference in treatment efficacy between PRS Regimens III, IV, V, and VI at 3 weeks (*p* > 0.75, One-way ANOVA with Tukey’s correction for multiple comparisons). In contrast, mice treated with the Standard Regimen had a much slower reduction in bacterial burden in their lungs and continued to have about 2 log CFU in their lungs after 6 weeks of treatment (Fig 3A and 3B and S10 Table). All four of the PRS regimens tested had significantly lower CFU burdens in the lungs at 3, 4, 5, and 6 weeks (Standard Regimen vs. PRS Regimens, *p* < 0.0001, One-way ANOVA with Tukey’s correction for multiple comparisons). Overall, lung appearance correlated with treatment efficacy. Sham-treated mice had numerous large and distinct lesions on the surfaces of their lungs. Mice treated with the Standard Regimen had smaller and fewer lung lesions. Mice treated with PRS Regimens III, IV, V or VI barely had any lesions on the surfaces of their lungs (S2 Fig).

### Treatment with PRS Regimens III–VI provides ultra-rapid relapse-free cure

We infected BALB/c mice (10 per group) by aerosol with virulent *M*. *tuberculosis*, Erdman strain. Two weeks later, we treated the mice with the Standard Regimen, PRS Regimen III, or one of the three new PRS Regimens (IV, V, or VI) 5 days per week for 3, 4, 5 or 6 weeks. After 3 months rest without treatment, the mice were euthanized and the bacterial burden in the lungs was measured by plating their entire lungs for CFU. We defined relapse as the presence of 1 or more *M*. *tuberculosis* CFU in their lungs. As shown in Table 4 and S11B, S11D and S11E Tables, PRS Regimen III, V, and VI achieved relapse-free cure after only 3 weeks of treatment. In mice treated with PRS Regimen IV, after 3 weeks of treatment, seven out of nine mice had no *M*. *tuberculosis* CFU in their lungs, and after 4 weeks of treatment, 9/10 mice had no CFU in their lungs and the 10^th^ mouse had only a single CFU. Mice treated with PRS Regimen IV achieved relapse-free cure after 5 weeks of treatment (Table 4 and S11C Table). In contrast, all 10 of the mice treated with the Standard Regimen for 6 weeks followed by a 3-month rest continued to have high levels of CFU in their lungs (S11A Table).

**Table 4:** Assessment of relapse^a^.

Treatment duration (wk)	Standard Regimen^b^	PRS Regimen^c^			
		III	IV	V	VI
3	—	0/10 (0%)	2/9 (22%)	0/10 (0%)	0/10 (0%)
4	—	0/10 (0%)	1/10 (10%)	0/10 (0%)	0/10 (0%)
5	—	0/10 (0%)	0/9 (0%)	0/10 (0%)	0/10 (0%)
6	10/10 (100%)	—	0/10 (0%)	0/7 (0%)	0/10 (0%)

^a^Two weeks after aerosol infection with *M*. *tuberculosis*, mice were treated 5 days per week for the duration indicated in the leftmost column. Three months after treatment cessation, mice were euthanized to assess relapse by plating out the entire lung homogenate. Shown are the number of mice with *M*. *tuberculosis* CFU detected in the lung over the total number of mice assessed for the treatment duration indicated and in parenthesis the percentage of mice that relapsed.

^b^Standard Regimen is comprised of INH, RIF, EMB and PZA at 25, 10, 100 and 150 mg/kg, respectively.

^c^PRS Regimen III is comprised of CFZ, BDQ, PZA and SQ109 at 25, 30, 450 and 25 mg/kg, respectively. PRS Regimen IV is comprised of CFZ, BDQ, PZA and AC at 25, 37, 50 and 66.7–16.7 mg/kg, respectively. PRS Regimen V is comprised of CFZ, BDQ, PZA and DLM at 25, 40, 185 and 0.83 mg/kg, respectively. PRS Regimen VI is comprised of CFZ, BDQ and PZA at 25, 40 and 185 mg/kg, respectively.

## Discussion

We have demonstrated two new TB treatment regimens, one comprising 4 drugs (CFZ, BDQ, PZA, and DLM–PRS Regimen V) and one comprising 3 drugs (CFZ, BDQ, and PZA–PRS Regimen VI) that achieve relapse-free cure in the BALB/c mouse model with only 3 weeks of treatment, and a fourth regimen (CFZ, BDQ, PZA, and AC, PRS Regimen IV) that does so in 5 weeks. The 4-drug regimens should be effective against MDR-TB because the only first line drug utilized is PZA. In addition, because these regimens do not include a fluoroquinolone, or an aminoglycoside or other injectable agent, they should also be effective against many cases of XDR-TB. While PRS Regimen IV was two weeks slower to achieve relapse-free cure than PRS Regimens III, V, and VI, this difference was relatively minor, since only two out of 10 PRS Regimen IV mice had detectable CFU after 3 weeks treatment and only one out of 10 had a single CFU at 4 weeks.

PRS Regimens V and VI employ the same doses of CFZ, BDQ, and PZA and differ only in that PRS Regimen V includes DLM whereas PRS Regimen VI does not. Nevertheless, these two regimens show identical EBA_14_ and lung sterilizing efficacy, and both achieve relapse-free cure in the mouse BALB/c model with only three weeks of treatment. While these two regimens are equally effective in lung sterilization and time required to achieve relapse-free cure, the inclusion of DLM may ultimately be useful in helping to prevent the development of drug resistance in patients with MDR-TB or XDR-TB.

Various strains of *M*. *tuberculosis* exhibit different rates of growth *in vitro* [38] and in mice [39], with some clinical isolates showing faster initial growth in the lungs of mice than the Erdman strain [40]. We anticipate that PRS Regimens IV, V and VI will show comparable efficacy against strains with faster doubling times, as faster growing strains may be more rapidly susceptible to killing by cidal antibiotics, but further animal studies would be needed to explore the interplay between *in vitro*/*in vivo* mycobacterial strain growth rates and the efficacy of antibiotic regimens.

The dose levels of all the drugs used in the new PRS regimens are readily achievable in humans. For BDQ, the dose in mice in PRS Regimens V and VI is 40 mg/kg administered 5 days/week. Mice dosed with BDQ at 15, 30, and 60 mg/kg 5 days/week have AUCs of 12, 21, and 48 μg h/ml, respectively [41]. Since BDQ shows linear pharmacokinetics in both mice and humans, the PRS Regimens V and VI dose in mice would yield an AUC of ~32 μg h/ml in mice. Humans administered BDQ at a dose of 400 mg daily for 1 week have an AUC of 64.75 μg h/ml [42]. Hence, a daily human dose of 200 mg/day is roughly equivalent to the optimized mouse dose in our study for PRS Regimens V and VI. Current clinical practice for BDQ is to initiate dosing at 400 mg once daily for two weeks, followed by 200 mg three times/week, which equates to an average daily dose over the first 4 weeks of 243 mg/day. Therefore, our estimated human equivalent dose of 200 mg/day for PRS Regimens V and VI is close to, but somewhat lower than, the current standard human dose.

For PZA, the dose in mice for PRS Regimens V and VI is 185 mg/kg (daily in the EBA_14_ study and 5 days/week in the efficacy study), which is only slightly higher than the 150 mg/kg dose of PZA that is commonly used in mouse TB treatment studies. Body surface area based dose conversion guidance provided by the FDA (“FDA Guidance for Industry: Estimating the Maximum Safe Starting Dose in Initial Clinical Trials for Therapeutics in Adult Healthy Volunteers”, available at https://www.fda.gov/regulatory-information/search-fda-guidance-documents/estimating-maximum-safe-starting-dose-initial-clinical-trials-therapeutics-adult-healthy-volunteers) indicates that a daily dose of 185 mg/kg in the mouse corresponds to ~15 mg/kg in humans, which is at the lower end of the 15–30 mg/kg dosing recommendations for PZA provided in the CDC–ATS guidelines and hence readily achievable.

For CFZ, the dose in mice for PRS Regimens IV, V, and VI is 25 mg/kg, which is roughly equivalent to a human daily dose of 2 mg/kg based on the FDA body surface area dose conversion guidance. The dose of CFZ used in current clinical practice for lepromatous leprosy complicated by erythema nodosum leprosum is 100 mg to 200 mg daily for up to 3 months, with dose tapering down to 100 mg after that 3-month period [43]. Thus, the estimated human CFZ daily dose for our regimens (2 mg/kg), which for a 60 kg individual would equate to a human dose of 120 mg daily, is readily achievable and consistent with clinical dosing used for leprosy, though treatment for TB would be of much shorter duration.

For DLM, the dose for mice in PRS Regimen V is 0.83 mg/kg. An estimate of the human equivalent dose is best based upon pharmacokinetic data in mice and humans since these data indicate that the FDA guidance would underestimate the human equivalent dose. Repeated daily dosing of DLM in mice at 30 mg/kg yields an AUC_0-24h_ of 36.5 μg h/ml [44]. Since DLM exhibits linear PK, repeated dosing at 0.83 mg/kg as in PRS Regimen V would yield an AUC_0-24h_ of ~ 1 μg h/ml in mice. Repeated daily dosing of DLM in humans at the currently recommended dose of 100 mg BID yields an AUC_0-24h_ of 7.9 μg h/ml [44]; hence the human equivalent dose for 0.83 mg/kg in mice would be ~25 mg/day (1/7.9 x 200 mg/day), a relatively low and readily achievable dose. In clinical trials, DLM has been well tolerated and its serious adverse event profile has been similar to that of placebo, except for QT prolongation [45]. The effect on QT prolongation when DLM is combined with other QT-prolonging drugs, such as BDQ, is the subject of ongoing clinical trials (e.g. ACTG A5343), but thus far appears to be predictable and manageable [46,47].

For AC, our optimized dose for PRS Regimen IV is 66.7/16.7 mg/kg. A body surface area based calculation indicates that this dose corresponds to a human dose of ~5.4 mg/kg daily or ~325 mg daily for a 60 Kg person. In patients with MDR-TB, much higher doses of 1000 mg/250 mg have been administered three times daily. Hence, our estimated human dose is readily achievable.

Current WHO guidance for treatment of rifampicin-resistant or MDR-TB recommends a regimen with at least five effective TB drugs during the intensive phase, including pyrazinamide and four core second line drugs, one chosen from Group A (levofloxacin, MXF, gatifloxacin), one from Group B (amikacin, capreomycin, kanamycin, streptomycin), and at least two from Group C (ethionamide, PRO, CYS, LZD, CFZ). However, these recommendations are based on experience with combinations of less effective drugs and drug combinations that may have strong antagonistic interactions. For example, our PRS screening rapidly eliminated LZD and MXF because of their antagonistic interactions. PRS Regimens III–V have only 4 drugs and PRS Regimen VI has only three drugs. Whether our PRS regimens will be as effective in achieving ultra-rapid relapse-free cure in mice infected with drug-resistant TB as drug-sensitive TB is an important question that will require further investigation. If resistance mutations do not otherwise alter the bacterial phenotype, then our PRS regimens should be as effective against drug-resistant TB (except perhaps for those with PZA resistance), as drug sensitive TB. On the other hand, resistance mutations may be accompanied by compensatory mutations that further alter the bacterial phenotype. Indeed, because the evolution of MDR-TB is highly heterogeneous [48], a result obtained with a single strain, e.g. a RIF-resistant *M*. *tuberculosis*, may not accurately predict the outcome with other drug-resistant strains. Ultimately, as suggested in a recent analysis of clinical trials of shorter TB treatment regimens [49], the appropriate duration of treatment may require a stratified approach, or a “personalized medicine approach”, that considers both bacterial and host factors, rather than a “one-size-fits-all” approach.

Our data demonstrate that PRS Regimens II–VI have the potential to greatly shorten the duration of treatment of both drug-sensitive and drug-resistant TB. In a previous study, we found that PRS Regimen III reduced the time to achieve relapse-free cure in mice by 80% vs. the Standard Regimen. The current study demonstrates that PRS Regimens V and VI are equivalent to our benchmark PRS Regimen III in the speed with which they achieve relapse-free cure and PRS Regimen IV is nearly equivalent. If clinical tests replicate the results of our animal studies, allowing treatment time to be reduced by ~80%, then use of these new regimens has the potential to be a game changer in the field of TB treatment, as dramatically shorter treatment should increase compliance and treatment completion and greatly decrease the burden of TB treatment on resource poor health care systems.

## Supporting information

S1 Table Drug concentrations used in macrophage infection model of *M*. *tuberculosis*. (PDF)

S2 Table Screening test two-level orthogonal array experimental design and experimental results. (PDF)

S3 Table Iteration 1, three-level orthogonal array central composite design and experimental results. (PDF)

S4 Table Iteration 2, three-level orthogonal array central composite design and experimental results. (PDF)

S5 Table Iteration 3A, five-level orthogonal array central composite design and experimental results. (PDF)

S6 Table Iteration 3B, five-level orthogonal array central composite design and experimental results. (PDF)

S7 Table Iteration 3C, three-level orthogonal array central composite design and experimental results. (PDF)

S8 Table *In vivo* treatment efficacy of the 14 top 4-drug experimental regimens identified from studies in *M*. *tuberculosis* infected THP-1 macrophages using the PRS platform. (PDF)

S9 Table Mouse lung burden of *M*. *tuberculosis* in PRS Regimens IV and V optimal dose finding study. (PDF)

S10 Table Mouse lung burden of *M*. *tuberculosis* in treatment efficacy study. (PDF)

S11 Table Mouse lung burden of *M*. *tuberculosis* in relapse study. (PDF)

S1 Fig Dose-response curve of delamanid (DLM). (PDF)

S2 Fig Lung pathology in mice upon completion of 3, 4, and 5 weeks of treatment. (PDF)
