# Correction: Artificial intelligence enabled parabolic response surface platform identifies ultra-rapid near-universal TB drug treatment regimens comprising approved drugs

**DOI:** 10.1371/journal.pone.0217670

**Published:** 2019-05-30

**Authors:** The PLOS ONE Staff

The ORCID iD is missing for the corresponding author. The publisher apologizes for the error. Author Marcus A. Horwitz’s ORCID iD is: 0000-0001-6525-7147 (https://orcid.org/0000-0001-6525-7147).

In Table 2, Prothionamide was mistakenly abbreviated “PRS” instead of “PRO” in the second column, “Screening Test”. Please see the corrected Table 2 here.

**Table 2:** Screening of combinatorial drugs in macrophage model of *M*. *tuberculosis* infection.

	Screening test	Iteration #1	Iteration #2	Iteration #3A	Iteration #3B	Iteration #3C
Effect levels^a^	2	3	3	5	5	3
Test runs^b^	150	102	155	75	75	82
Drugs^c^	AC	AC	AC	AC	AC	AC
	BDQ	BDQ	BDQ	BDQ	BDQ	BDQ
	CFZ	CFZ	CFZ	CFZ	CFZ	CFZ
	DLM	DLM	DLM	DLM	DLM	DLM
	RIF	RIF	RIF	RIF	RIF	RIF
	SQ109	SQ109	SQ109	SQ109	SQ109	SQ109
	PA824	PA824	PA824	PA824		PA824
	PZA	PZA	PZA	PZA		
	EMB	EMB	EMB			
	PAS	PAS				
	PRO	PRO				
	CYS					
	INH					
	LZD					
	MXF					

^a^Screening test was conducted at individual drug concentration that yielded 0% or 10% of the maximal inhibition to the IPTG-induced green fluorescence signal. Iteration #1 was conducted at individual drug concentration that gave 0% or 10% of the maximal inhibition level and one-half of the concentration that gave 10% of the maximal inhibition level. Iteration #2 was done at 0% or 15% effect levels and one-half of the concentration that gave 15% of the maximal inhibition level. Iterations #3A and 3B were conducted at 0% or 20% effect level and two-thirds, one-half or one-fourth of the concentration that gave 20% of the maximal inhibition level. Iteration #3C was conducted at 0% or 15% effect levels and one-half of the concentration that gave 15% of the maximal inhibition level.

^b^Number of combinatorial drug test runs in each experiment.

^c^List of drugs tested at screening and each stage of iterations.

On the right side of Fig 1, Group O was mistakenly omitted from the alphabetical list of drug regimen definitions. Please see the corrected Fig 1 here.

**Fig 1:**
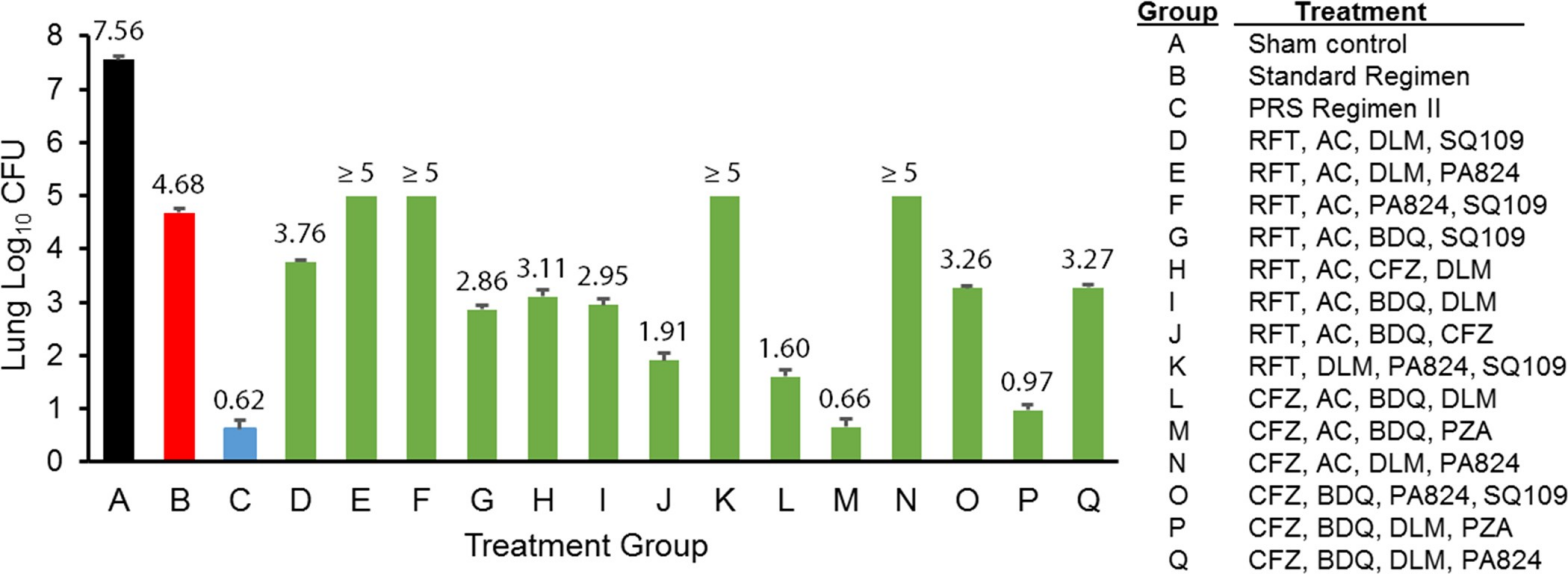
*In vivo* short-term efficacy screen of experimental regimens identified in macrophage studies using the PRS platform. *M*. *tuberculosis* infected mice were sham treated (Treatment Group A) or treated with the Standard Regimen (Treatment Group B), PRS Regimen II (Treatment Group C) or one of the top 4-drug combinations (Treatment Groups D-Q) identified from macrophage screening using the PRS platform starting from a pool of 15 TB drugs 5 days per week for 3 weeks. Three days after the last treatment, mice were euthanized to determine bacterial number in the lung. Standard Regimen is comprised of INH, RIF, EMB and PZA at 25, 10, 100 and 150 mg/kg, respectively. PRS Regimen II is comprised of CFZ, BDQ, PZA and EMB at 25, 30, 450 and 100 mg/kg, respectively. Drug doses used in the top 4-drug experimental regimens are as follows: 200–50 mg/kg for AC, 30 mg/kg for BDQ, 25 mg/kg for CFZ, 2.5 mg/kg for DLM, 100 mg/kg for PA824, 450 mg/kg for PZA, 10 mg/kg for RPT and 25 mg/kg for SQ109.
