# Supplementary material for: Artificial intelligence enabled parabolic response surface platform identifies ultra-rapid near-universal TB drug treatment regimens comprising approved drugs
Source: PLoS One. 2019 May 10;14(5):e0215607. doi: 10.1371/journal.pone.0215607 (PMC6510528; doi:10.1371/journal.pone.0215607)
Supplement: S1 Table — (PDF) [file pone.0215607.s001.pdf]

S1 Table. Drug concentrations used in macrophage infection model of *M. tuberculosis*.

Drug[a]	Screening test 10% level[b]	Iteration 1 10% level	Iteration 2 15% level	Iteration 3A 20% level	Iteration 3B 20% level	Iteration 3C 15% level
**AC**	4.00	3.60	3.00	3.62	3.62	5.34
**CFZ**	0.025	0.05	0.127	0.14	0.14	0.051
**CYC**	5.00					
**EMB**	0.05	0.075	0.20	0.22	0.22	0.20
**INH**	0.006	0.012	0.020	0.023	0.023	0.023
**LZD**	0.02					
**MXF**	0.02					
**PA824**	0.00132	0.0025	0.0032	0.00368		0.00261
**PAS**	0.05	0.10				
**PRO**	0.0133	0.02				
**PZA**	17.00	17.00	22.00	24.50	24.50	20.70
**RIF**	0.005	0.0065	0.016	0.0176	0.0176	0.00929
**SQ109**	0.066	0.16	0.217		0.24	0.19254
**BDQ**	0.0066	0.008	0.007	0.00884	0.00884	0.01076
**DLM**	0.0006	0.0008	0.001	0.00117	0.00117	0.00134

[a]All drug concentrations shown are in µg/ml.

[b]Shown are concentration of individual drugs used in the screening test or iterations to give the indicated percentage of inhibition of the maximal IPTG-induced green fluorescence level in the macrophage infection model of Mtb-iGFP.
