# Supplementary material for: Artificial intelligence enabled parabolic response surface platform identifies ultra-rapid near-universal TB drug treatment regimens comprising approved drugs
Source: PLoS One. 2019 May 10;14(5):e0215607. doi: 10.1371/journal.pone.0215607 (PMC6510528; doi:10.1371/journal.pone.0215607)
Supplement: S2 Table — (PDF) [file pone.0215607.s002.pdf]

S2 Table. Screening test two-level orthogonal array experimental design and experimental results.

Run/Drug	AC	CFZ	CYC	EMB	INH	LZD	MXF	PA824	PAS	PRO	PZA	RIF	SQ109	BDQ	DLM	% Inhibition Mean	N	SE
Control 1 (no infection control)																97%	18	0.3%
Control 2 (no IPTG control)																93%	18	0.5%
Control 3 (no drug control)																-1%	18	5.4%
Control 4 (all drug control, at 10% drug effect level)																61%	9	1.6%
1	2	2	2	1	2	2	2	2	1	1	1	1	1	1	1	25%	3	3.6%
2	2	1	1	1	1	1	2	2	1	2	2	2	2	1	1	31%	3	2.2%
3	1	1	2	1	2	1	1	2	2	2	1	1	2	2	1	28%	3	1.5%
4	2	2	2	2	2	2	2	2	2	2	2	2	2	2	2	56%	3	3.4%
5	1	2	1	2	1	2	1	2	1	2	1	1	2	1	2	58%	3	1.4%
6	1	2	2	2	1	1	2	1	1	2	1	2	1	2	1	38%	3	3.0%
7	1	2	2	1	1	1	2	1	2	1	2	1	2	1	2	10%	3	3.4%
8	1	1	1	1	2	2	2	1	2	2	1	2	1	1	2	17%	3	3.6%
9	2	1	2	1	1	2	1	1	1	2	2	1	1	2	2	29%	3	8.4%
10	1	2	1	1	1	2	1	2	2	1	2	2	1	2	1	28%	3	5.1%
11	2	1	2	2	1	2	1	1	2	1	1	2	2	1	1	48%	3	2.1%
12	2	2	1	1	2	1	1	1	1	1	1	2	2	2	2	32%	3	5.7%
13	2	2	1	2	2	1	1	1	2	2	2	1	1	1	1	57%	3	1.7%
14	1	1	2	2	2	1	1	2	1	1	2	2	1	1	2	51%	3	3.3%
15	2	1	1	2	1	1	2	2	2	1	1	1	1	2	2	59%	3	3.0%
16	1	1	1	2	2	2	2	1	1	1	2	1	2	2	1	43%	3	4.5%
17	1	2	2	2	1	2	2	2	1	1	2	2	2	1	1	26%	3	2.3%
18	1	2	1	1	1	1	1	1	2	2	1	2	2	1	1	3%	3	5.1%
19	2	2	1	1	2	2	1	2	1	2	2	2	1	1	2	62%	3	0.7%
20	2	2	2	2	2	1	2	1	2	1	1	2	1	1	2	55%	3	0.9%
21	2	1	1	1	1	2	2	1	1	1	1	2	1	2	1	42%	3	2.5%
22	2	2	1	2	2	2	1	2	2	1	1	1	2	2	1	57%	3	1.5%
23	1	1	1	1	2	1	2	2	2	1	2	2	2	2	2	46%	3	1.4%
24	1	1	2	2	2	2	1	1	1	2	1	2	2	2	2	49%	3	2.1%
25	2	1	2	1	1	1	1	2	1	1	1	1	2	1	2	41%	3	1.0%
26	1	2	2	1	1	2	2	2	2	2	1	1	1	2	2	25%	3	6.0%
27	1	1	1	2	2	1	2	2	1	2	1	1	1	1	1	28%	3	3.7%
28	2	1	2	2	1	1	1	2	2	2	2	2	1	2	1	53%	3	3.2%
29	1	1	2	1	2	2	1	1	2	1	2	1	1	1	1	-2%	3	6.6%
30	2	1	1	2	1	2	2	1	2	2	2	1	2	1	2	33%	3	3.9%
31	1	2	1	2	1	1	1	1	1	1	2	1	1	2	2	52%	3	2.0%
32	2	2	2	1	2	1	2	1	1	2	2	1	2	2	1	35%	3	1.7%
33	1	1	1	2	1	2	1	1	1	2	2	2	1	1	1	36%	3	4.3%
34	2	2	2	2	1	2	1	2	2	1	2	1	1	1	2	52%	3	2.5%
35	1	2	1	1	2	2	2	2	2	2	2	1	2	1	1	29%	3	2.1%
36	1	2	2	1	2	1	1	1	2	2	2	2	1	2	2	27%	3	1.0%
37	1	1	1	1	1	2	1	1	2	1	1	1	2	2	2	23%	3	2.4%
38	2	1	1	2	2	1	1	2	2	2	1	2	2	1	2	72%	3	0.7%
39	1	2	1	2	2	2	2	2	1	1	1	2	1	2	2	70%	3	1.6%
40	1	1	2	2	1	1	2	2	1	2	2	1	2	2	2	43%	3	2.2%
41	2	2	2	1	1	2	1	2	1	2	1	2	2	2	1	26%	3	4.8%
42	2	1	2	1	2	2	2	1	1	1	2	2	2	1	2	49%	3	2.3%
43	1	1	2	1	1	1	2	2	2	1	1	2	1	1	1	13%	3	1.8%
44	2	2	1	1	1	1	2	1	1	2	1	1	1	1	2	40%	3	3.0%
45	2	1	1	1	2	1	1	2	1	1	2	1	1	2	1	43%	3	4.7%
46	1	2	2	2	2	1	1	1	1	1	1	1	2	1	1	12%	3	2.8%
47	2	2	1	2	1	1	2	1	2	1	2	2	2	2	1	56%	3	1.8%
48	2	1	2	2	2	2	2	1	2	2	1	1	1	2	1	20%	3	9.3%
49	1	2	1	2	2	2	1	1	2	1	2	2	2	1	2	59%	3	1.8%
50	2	1	1	1	2	1	2	1	2	1	1	1	2	1	1	6%	3	4.8%
51	2	2	2	2	1	2	2	1	1	1	1	1	2	2	2	57%	3	0.7%
52	2	1	2	2	2	2	1	2	1	2	2	1	2	1	1	30%	3	3.2%
53	1	1	1	1	1	2	2	2	1	1	2	1	1	1	2	15%	3	3.9%
54	1	2	1	1	2	2	1	1	1	2	1	1	1	2	1	34%	3	0.9%
55	2	1	1	2	2	1	2	1	1	2	2	2	1	2	2	75%	3	1.7%
56	1	2	2	1	2	1	2	2	1	2	1	2	2	1	2	25%	3	1.4%
57	2	2	1	2	1	1	1	2	1	1	1	2	1	1	1	61%	3	3.3%
58	1	2	2	2	2	1	2	2	2	1	2	1	1	2	1	40%	3	3.8%
59	2	2	2	1	1	2	2	1	2	2	2	2	1	1	1	20%	3	2.2%
60	1	1	2	2	1	1	1	1	2	2	1	1	1	1	2	27%	3	1.7%
61	1	1	2	1	1	1	1	1	1	1	2	2	2	2	1	4%	3	5.2%
62	1	1	1	2	1	2	2	2	2	2	1	2	2	2	1	57%	3	0.4%
63	2	1	2	1	2	2	1	2	2	1	1	2	1	2	2	49%	3	1.7%
64	2	2	1	1	1	1	1	2	2	2	2	1	2	2	2	62%	3	2.1%
65	1	1	1	1	2	2	1	2	1	1	1	2	2	1	1	13%	3	3.4%

**66**	2	1	2	1	1	2	2	2	2	1	2	1	2	2	1	39%	3	3.3%
**67**	1	2	1	1	1	2	2	1	1	2	2	2	2	2	2	47%	3	1.1%
**68**	2	2	2	1	2	2	1	1	2	2	1	1	2	1	2	31%	3	5.9%
**69**	2	1	1	1	1	1	1	1	2	1	2	2	1	1	2	48%	3	1.8%
**70**	1	1	2	1	2	1	2	1	1	1	1	1	1	2	2	14%	3	9.5%
**71**	1	2	1	2	1	2	2	1	2	1	1	1	1	1	1	33%	3	3.2%
**72**	2	2	1	1	2	1	2	2	2	2	1	2	1	2	1	32%	3	2.3%
**73**	1	1	1	2	2	2	1	2	2	2	2	1	1	2	2	59%	3	1.2%
**74**	2	2	1	2	2	1	2	2	1	1	2	1	2	1	2	70%	3	2.2%
**75**	1	2	2	1	1	1	1	2	1	2	2	1	1	1	1	12%	3	5.2%
**76**	2	1	1	2	1	1	1	1	1	2	1	1	2	2	1	39%	3	1.3%
**77**	1	1	2	2	2	1	2	1	2	2	2	2	2	1	1	32%	3	3.6%
**78**	1	2	2	2	1	1	1	2	2	1	1	2	2	2	2	61%	3	1.4%
**79**	2	2	2	2	2	2	1	1	1	1	2	2	1	2	1	51%	3	2.9%
**80**	2	1	2	2	1	2	2	2	1	2	1	2	1	1	2	75%	3	1.0%
**81**	2	1	1	2	2	2	2	2	2	1	2	2	1	1	1	63%	3	1.4%
**82**	1	2	1	2	2	1	1	2	1	2	2	2	2	2	1	49%	3	3.3%
**83**	1	1	1	2	1	1	2	1	1	1	1	2	2	1	2	63%	3	1.0%
**84**	2	2	2	2	1	1	2	2	2	2	1	1	2	1	1	44%	3	5.1%
**85**	2	1	1	1	2	2	2	2	1	2	1	1	2	2	2	60%	3	1.4%
**86**	1	2	2	1	2	2	2	1	2	1	1	2	2	2	1	11%	3	2.3%
**87**	2	1	2	1	2	1	1	1	1	2	1	2	1	1	1	32%	3	1.3%
**88**	2	1	2	2	2	1	1	1	2	1	2	1	2	2	2	45%	3	4.5%
**89**	2	2	1	2	1	2	1	1	2	2	1	2	1	2	2	63%	3	3.3%
**90**	1	1	2	1	1	2	1	2	2	2	2	2	2	1	2	49%	3	0.9%
**91**	2	2	1	1	1	2	1	1	1	1	2	1	2	1	1	40%	3	2.3%
**92**	1	2	1	1	2	1	1	2	2	1	1	1	1	1	2	39%	3	1.1%
**93**	1	1	1	1	1	1	2	1	2	2	2	1	1	2	1	16%	3	12.0%
**94**	2	2	2	1	1	1	2	2	1	1	2	2	1	2	2	40%	3	1.0%
**95**	1	1	2	2	1	2	1	2	1	1	1	1	1	2	1	41%	3	1.0%
**96**	1	2	2	2	2	2	2	1	1	2	2	1	1	1	2	34%	3	4.7%
**97**	1	2	1	1	1	1	2	2	1	1	1	1	2	2	1	27%	3	4.0%
**98**	2	2	1	2	2	2	2	1	1	2	1	2	2	1	1	45%	3	0.3%
**99**	1	1	2	2	2	2	2	2	2	1	1	1	2	1	2	41%	3	3.1%
**100**	2	2	1	1	2	2	2	1	2	1	2	1	1	2	2	58%	3	1.4%
**101**	2	1	1	2	1	2	1	2	1	1	2	2	2	2	2	80%	3	1.0%
**102**	1	2	2	1	1	2	1	1	1	1	1	2	1	1	2	3%	3	2.0%
**103**	2	1	2	2	1	1	2	1	1	1	2	1	1	1	1	39%	3	3.2%
**104**	1	1	1	2	2	1	1	1	2	1	1	2	1	2	1	50%	3	2.0%
**105**	2	1	2	1	1	1	2	1	2	2	1	2	2	2	2	41%	3	4.0%
**106**	2	1	1	1	1	2	1	2	2	2	1	1	1	1	1	30%	3	3.2%
**107**	2	2	2	2	2	1	1	2	1	2	1	1	1	2	2	58%	3	0.3%
**108**	1	2	2	2	1	2	1	1	2	2	2	1	2	2	1	46%	3	2.7%
**109**	1	1	1	1	2	1	1	1	1	2	2	1	2	1	2	21%	3	2.3%
**110**	1	2	1	2	1	1	2	2	2	2	2	2	1	1	2	69%	3	1.1%
**111**	1	1	2	1	2	2	2	2	1	2	2	2	1	2	1	4%	3	9.8%
**112**	2	2	2	1	2	1	1	2	2	1	2	2	2	1	1	27%	3	3.1%
**113**	2	2	2	1	1	1	1	1	2	1	1	1	1	2	1	33%	3	4.0%
**114**	1	1	1	1	1	1	1	2	1	2	1	2	1	2	2	52%	3	3.3%
**115**	2	1	2	2	2	1	2	2	1	1	1	2	2	2	1	57%	3	1.3%
**116**	1	1	1	2	1	1	1	2	2	1	2	1	2	1	1	29%	3	4.7%
**117**	2	2	2	2	1	1	1	1	1	2	2	2	2	1	2	45%	3	2.9%
**118**	1	1	2	2	1	2	2	1	2	1	2	2	1	2	2	48%	3	3.2%
**119**	1	2	1	1	2	1	2	1	1	1	2	2	1	1	1	6%	3	3.2%
**120**	1	2	1	2	2	1	2	1	2	2	1	1	2	2	2	52%	3	5.1%
**121**	1	2	2	2	2	2	1	2	2	2	1	2	1	1	1	33%	3	1.6%
**122**	2	1	1	1	2	2	1	1	2	2	2	2	2	2	1	47%	3	3.3%
**123**	1	2	2	1	2	2	1	2	1	1	2	1	2	2	2	40%	3	3.9%
**124**	1	1	2	1	1	2	2	1	1	2	1	1	2	1	1	-13%	3	2.7%
**125**	2	2	1	1	1	2	2	2	2	1	1	2	2	1	2	54%	3	2.6%
**126**	2	1	2	1	2	1	2	2	2	2	2	1	1	1	2	31%	3	2.1%
**127**	2	1	1	2	2	2	1	1	1	1	1	1	1	1	2	58%	3	1.9%
**128**	2	2	1	2	1	2	2	2	1	2	2	1	1	2	1	60%	3	0.7%
**129**	1	1	2	2	1	1	2	1	2	1	2	1	1	2	1	31%	3	0.6%
**130**	1	1	1	1	1	1	2	1	1	2	2	2	2	2	1	25%	3	1.1%
**131**	1	1	1	1	1	1	1	1	2	2	2	1	2	1	1	21%	3	2.7%
**132**	1	1	1	2	2	1	1	2	2	2	2	1	2	2	1	47%	3	1.7%
**133**	1	2	1	2	1	2	1	1	2	2	1	2	1	1	1	48%	3	2.0%
**134**	1	1	1	1	1	1	2	2	1	1	1	1	2	1	2	19%	3	9.7%
**135**	1	1	2	2	2	1	2	1	2	1	2	1	2	2	2	39%	3	4.8%
**136**	2	2	2	1	1	1	2	1	2	2	1	2	1	1	2	24%	3	1.2%
**137**	1	2	1	1	2	2	1	2	1	2	1	2	2	2	2	48%	3	3.9%
**138**	2	2	1	2	2	1	1	2	2	1	2	1	2	1	1	48%	3	0.6%
**139**	1	2	1	1	1	1	1	1	2	1	2	2	1	1	2	32%	3	3.8%
**140**	1	2	1	2	1	2	2	2	1	1	1	2	2	2	1	49%	3	2.8%

**141**	2	1	1	1	2	1	2	2	2	2	1	1	2	1	1	33%	3	3.2%
**142**	1	1	2	2	2	2	1	1	1	1	2	1	1	1	2	31%	3	1.6%
**143**	2	1	1	1	2	1	1	1	2	1	2	2	1	2	1	27%	3	1.6%
**144**	2	1	1	2	1	1	1	1	2	1	1	2	2	1	2	71%	3	0.6%
**145**	2	2	1	2	1	1	1	2	1	1	1	2	2	1	2	74%	3	1.0%
**146**	1	1	2	1	2	1	2	2	2	2	1	2	1	1	2	19%	3	1.3%
**147**	2	2	2	1	2	1	2	2	2	2	2	1	2	2	1	37%	3	2.3%
**148**	1	1	2	1	2	1	2	2	2	1	2	1	2	2	1	32%	3	4.3%
**149**	1	1	2	1	2	2	1	1	2	1	1	2	2	2	1	21%	3	3.4%
**150**	2	1	2	2	1	2	2	2	1	2	1	1	1	1	1	29%	3	3.7%
**SR**																31%	3	0.3%

Drug dose “1” and “2” represent 0% and 10% of the drug effect level, respectively. Data shown are mean % inhibition, number of replicates (N), and standard error (SE). SR, Standard Regimen tested at 10% of the drug effect level.
