# Supplementary material for: Artificial intelligence enabled parabolic response surface platform identifies ultra-rapid near-universal TB drug treatment regimens comprising approved drugs
Source: PLoS One. 2019 May 10;14(5):e0215607. doi: 10.1371/journal.pone.0215607 (PMC6510528; doi:10.1371/journal.pone.0215607)
Supplement: S3 Table — (PDF) [file pone.0215607.s003.pdf]

S3 Table. Iteration 1, three-level orthogonal array central composite design and experimental results.

Run/Drug	AC	CFZ	EMB	PA824	PAS	PRO	PZA	RIF	SQ109	BDQ	DLM	% Inhibition		
												Mean	N	SE
Control 1 (no infection control)												98%	4	0.4%
Control 2 (no IPTG control)												98%	4	0.2%
Control 3 (no drug control)												0%	8	5.6%
Control 4 (all drug control, at 10% drug effect level)												90%	2	1.0%
1	2	2	3	1	3	1	3	1	1	1	2	59%	3	2.3%
2	1	2	1	1	3	1	1	3	2	2	1	15%	3	3.4%
3	3	2	2	2	1	1	3	1	2	2	3	83%	3	0.8%
4	2	3	3	1	1	3	3	3	2	2	3	86%	3	1.0%
5	1	1	1	2	3	2	2	3	3	3	3	80%	3	1.0%
6	1	2	1	2	1	1	2	2	1	1	1	24%	3	6.1%
7	2	1	3	1	2	2	3	2	3	3	1	54%	3	1.0%
8	1	3	1	1	1	3	1	2	3	3	2	57%	3	1.2%
9	2	2	3	3	2	1	2	2	2	2	2	67%	3	3.2%
10	1	2	1	3	2	1	3	1	3	3	1	58%	3	0.6%
11	1	3	1	3	3	3	3	3	1	1	2	68%	3	2.4%
12	1	3	1	2	2	3	2	1	2	2	2	49%	3	4.1%
13	3	2	2	3	2	1	1	3	1	1	3	94%	3	0.4%
14	3	1	2	1	2	2	2	3	2	2	2	64%	3	3.0%
15	3	2	2	1	3	1	2	2	3	3	3	78%	3	1.4%
16	3	1	2	3	1	2	1	1	3	3	2	73%	3	2.3%
17	1	1	1	3	1	2	3	2	2	2	3	82%	3	1.2%
18	3	3	2	3	3	3	1	2	2	2	1	57%	3	2.7%
19	2	2	3	2	1	1	1	3	3	3	2	79%	3	1.0%
20	3	1	2	2	3	2	3	2	1	1	2	74%	3	1.2%
21	2	3	3	2	2	3	1	2	1	1	3	86%	3	1.7%
22	2	1	3	2	3	2	1	1	2	2	1	30%	3	2.7%
23	3	3	2	1	1	3	2	1	1	1	1	50%	3	3.4%
24	2	1	3	3	1	2	2	3	1	1	1	75%	3	0.4%
25	3	3	2	2	2	3	3	3	3	3	1	74%	3	0.9%
26	1	1	1	1	2	2	1	1	1	1	3	14%	3	8.5%
27	2	3	3	3	3	3	2	1	3	3	3	87%	3	1.2%
28	3	1	3	3	2	3	3	1	1	2	2	81%	3	1.1%
29	3	3	3	3	1	1	3	2	3	1	1	79%	3	1.2%
30	3	3	3	1	2	1	1	1	2	3	1	57%	3	3.7%
31	1	1	2	3	2	3	2	2	3	1	3	83%	3	0.8%
32	2	1	1	2	1	3	3	1	3	1	1	46%	3	4.0%
33	2	2	1	3	3	2	1	2	3	1	2	71%	3	1.7%
34	3	2	3	1	1	2	1	2	1	2	3	87%	3	0.5%
35	1	3	2	1	2	1	3	2	1	2	2	38%	3	6.7%
36	2	1	1	3	2	3	1	3	2	3	1	50%	3	1.9%
37	2	2	1	1	1	2	2	1	2	3	2	30%	3	4.4%
38	1	3	2	2	3	1	1	1	3	1	2	60%	3	1.6%
39	2	1	1	1	3	3	2	2	1	2	1	36%	3	3.8%
40	3	1	3	2	1	3	2	2	2	3	2	69%	3	0.6%
41	2	2	1	2	2	2	3	3	1	2	2	61%	3	1.6%
42	1	2	2	3	3	2	2	1	1	2	1	28%	3	12.1%
43	1	2	2	2	2	2	1	2	2	3	1	36%	3	2.3%
44	3	3	3	2	3	1	2	3	1	2	1	77%	3	1.6%
45	2	3	1	1	2	1	2	3	3	1	3	79%	3	1.7%
46	1	1	2	1	3	3	3	1	2	3	3	45%	3	1.9%
47	3	2	3	2	2	2	2	1	3	1	3	85%	3	1.0%
48	1	1	2	2	1	3	1	3	1	2	3	86%	3	2.5%
49	2	3	1	2	3	1	3	2	2	3	3	81%	3	0.6%
50	1	3	2	3	1	1	2	3	2	3	2	62%	3	0.7%
51	2	3	1	3	1	1	1	1	1	2	3	80%	3	3.4%
52	1	2	2	1	1	2	3	3	3	1	1	57%	3	0.7%
53	3	2	3	3	3	2	3	3	2	3	3	81%	3	0.9%
54	3	1	3	1	3	3	1	3	3	1	2	73%	3	1.5%
55	2	1	2	1	1	1	1	2	2	1	1	22%	3	6.1%
56	2	1	2	2	2	1	2	1	1	3	1	32%	3	3.7%
57	1	1	3	3	3	1	1	2	1	3	3	90%	3	1.1%
58	3	3	1	2	1	2	1	3	2	1	1	53%	3	2.6%
59	1	1	3	2	2	1	3	3	2	1	3	87%	3	1.7%
60	3	3	1	3	2	2	2	2	1	3	1	61%	3	2.1%
61	2	2	2	3	1	3	3	2	1	3	2	77%	3	1.8%
62	3	1	1	3	3	1	2	1	2	1	2	53%	3	2.0%
63	1	3	3	3	2	2	1	3	3	2	2	79%	3	1.3%
64	2	2	2	1	2	3	1	1	3	2	2	60%	3	1.4%
65	3	2	1	2	3	3	1	1	1	3	3	77%	3	3.3%

**66**	3	1	1	2	2	1	1	2	3	2	2	67%	3	1.4%
**67**	3	3	1	1	3	2	3	1	3	2	1	66%	3	2.5%
**68**	2	1	2	3	3	1	3	3	3	2	1	67%	3	1.3%
**69**	2	3	2	1	3	2	1	3	1	3	3	77%	3	1.9%
**70**	1	1	3	1	1	1	2	1	3	2	3	53%	3	6.4%
**71**	2	3	2	2	1	2	2	2	3	2	3	81%	3	1.0%
**72**	1	3	3	1	3	2	2	2	2	1	2	44%	3	4.6%
**73**	1	3	3	2	1	2	3	1	1	3	2	68%	3	1.8%
**74**	2	3	2	3	2	2	3	1	2	1	3	84%	3	2.1%
**75**	3	2	1	3	1	3	2	3	3	2	3	89%	3	0.6%
**76**	1	2	3	2	3	3	3	2	3	2	1	53%	3	2.2%
**77**	1	2	3	3	1	3	1	1	2	1	1	49%	3	1.2%
**78**	1	2	3	1	2	3	2	3	1	3	1	55%	3	0.3%
**79**	3	2	1	1	2	3	3	2	2	1	3	69%	3	1.7%
**80**	2	2	2	2	3	3	2	3	2	1	2	57%	3	4.6%
**81**	3	1	1	1	1	1	3	3	1	3	2	66%	3	3.3%
**82**	2	2	2	2	2	2	2	2	2	2	2	59%	3	0.6%
**83**	2	3	1	1	2	1	1	3	1	1	1	45%	3	3.9%
**84**	3	2	1	1	1	2	1	1	1	3	1	34%	3	7.4%
**85**	2	1	3	1	1	1	3	1	1	1	2	51%	3	2.7%
**86**	3	1	2	1	1	1	1	1	3	1	1	62%	3	2.4%
**87**	2	1	1	2	3	1	1	2	1	1	1	34%	3	5.7%
**88**	3	1	1	1	2	1	1	1	3	1	3	73%	3	3.2%
**89**	2	1	1	3	1	1	2	3	1	1	1	52%	3	3.0%
**90**	3	1	2	1	1	1	1	2	1	3	1	52%	3	3.9%
**91**	2	3	1	1	1	2	1	1	1	1	3	63%	3	3.1%
**92**	3	1	1	3	1	1	1	1	2	1	2	64%	3	1.6%
**93**	1	2	1	1	2	1	3	1	1	3	1	34%	3	6.3%
**94**	1	1	1	1	3	3	1	1	1	2	2	27%	3	2.2%
**95**	1	1	1	3	2	1	1	1	2	1	3	48%	3	3.3%
**96**	1	1	2	1	3	1	1	3	1	1	2	26%	3	5.4%
**97**	1	1	1	2	1	1	2	1	1	2	3	36%	3	2.2%
**98**	1	3	1	1	1	1	3	3	1	1	2	46%	3	5.1%
**99**	1	1	2	1	1	3	1	1	1	3	2	22%	3	3.8%
**100**	1	1	3	1	1	1	1	2	1	2	3	77%	3	1.0%
**101**	1	2	1	1	3	2	1	1	2	1	1	12%	3	5.8%
**102**	3	2	1	3	1	1	2	1	2	1	1	59%	3	1.1%
**SR**												55%	3	1.5%

Drug dose “1”, “2” and “3” represent 0%, 5% and 10% of the drug effect level, respectively. Data shown are mean % inhibition, number of replicates (N), and standard error (SE). SR, Standard Regimen tested at 10% of the drug effect level.
