# Supplementary material for: Artificial intelligence enabled parabolic response surface platform identifies ultra-rapid near-universal TB drug treatment regimens comprising approved drugs
Source: PLoS One. 2019 May 10;14(5):e0215607. doi: 10.1371/journal.pone.0215607 (PMC6510528; doi:10.1371/journal.pone.0215607)
Supplement: S4 Table — (PDF) [file pone.0215607.s004.pdf]

S4 Table. Iteration 2, three-level orthogonal array central composite design and experimental results.

										% Inhibition		
										Mean	N	SE
Control 1 (no infection control)										99%	6	0.1%
Control 2 (no IPTG control)										98%	7	0.1%
Control 3 (no drug control)										0%	8	6.4%
Control 4 (all drug control, at 15% drug effect level)										89%	3	0.5%
Run/Drug	AC	CFZ	EMB	PA824	PZA	RIF	SQ109	BDQ	DLM			
1	1	1	1	1	1	1	1	1	1	-8%	3	7.5%
2	1	1	1	1	1	1	3	1	3	57%	3	4.6%
3	1	1	1	1	1	3	1	1	3	84%	3	0.4%
4	1	1	1	1	1	3	3	1	1	80%	3	3.0%
5	1	1	1	1	3	1	1	3	1	34%	3	3.7%
6	1	1	1	1	3	1	3	3	3	67%	3	5.8%
7	1	1	1	1	3	3	1	3	3	85%	3	3.0%
8	1	1	1	1	3	3	3	3	1	80%	3	1.8%
9	1	1	1	3	1	1	1	3	1	47%	3	3.7%
10	1	1	1	3	1	1	3	3	3	79%	3	2.8%
11	1	1	1	3	1	3	1	3	3	95%	3	0.5%
12	1	1	1	3	1	3	3	3	1	81%	3	0.1%
13	1	1	1	3	3	1	1	1	1	31%	3	2.7%
14	1	1	1	3	3	1	3	1	3	83%	3	2.9%
15	1	1	1	3	3	3	1	1	3	94%	3	0.9%
16	1	1	1	3	3	3	3	1	1	88%	3	0.9%
17	1	1	3	1	1	1	1	3	3	62%	3	3.4%
18	1	1	3	1	1	1	3	3	1	51%	3	2.3%
19	1	1	3	1	1	3	1	3	1	93%	3	0.4%
20	1	1	3	1	1	3	3	3	3	93%	3	0.3%
21	1	1	3	1	3	1	1	1	3	69%	3	3.8%
22	1	1	3	1	3	1	3	1	1	50%	3	3.7%
23	1	1	3	1	3	3	1	1	1	91%	3	1.9%
24	1	1	3	1	3	3	3	1	3	92%	3	0.6%
25	1	1	3	3	1	1	1	1	3	83%	3	0.5%
26	1	1	3	3	1	1	3	1	1	61%	3	1.9%
27	1	1	3	3	1	3	1	1	1	96%	3	0.6%
28	1	1	3	3	1	3	3	1	3	97%	3	0.2%
29	1	1	3	3	3	1	1	3	3	85%	3	1.9%
30	1	1	3	3	3	1	3	3	1	76%	3	1.0%
31	1	1	3	3	3	3	1	3	1	94%	3	0.4%
32	1	1	3	3	3	3	3	3	3	97%	3	0.3%
33	1	3	1	1	1	1	1	3	3	65%	3	2.2%
34	1	3	1	1	1	1	3	3	1	72%	3	3.0%
35	1	3	1	1	1	3	1	3	1	47%	3	1.7%
36	1	3	1	1	1	3	3	3	3	83%	3	0.7%
37	1	3	1	1	3	1	1	1	3	83%	3	1.2%
38	1	3	1	1	3	1	3	1	1	74%	3	3.2%
39	1	3	1	1	3	3	1	1	1	66%	3	2.2%
40	1	3	1	1	3	3	3	1	3	89%	3	1.5%
41	1	3	1	3	1	1	1	1	3	89%	3	0.8%
42	1	3	1	3	1	1	3	1	1	78%	3	2.5%
43	1	3	1	3	1	3	1	1	1	52%	3	2.3%
44	1	3	1	3	1	3	3	1	3	91%	3	0.3%
45	1	3	1	3	3	1	1	3	3	88%	3	1.0%
46	1	3	1	3	3	1	3	3	1	85%	3	1.2%
47	1	3	1	3	3	3	1	3	1	80%	3	1.4%
48	1	3	1	3	3	3	3	3	3	88%	3	1.2%
49	1	3	3	1	1	1	1	1	1	72%	3	1.8%
50	1	3	3	1	1	1	3	1	3	83%	3	0.7%
51	1	3	3	1	1	3	1	1	3	94%	3	1.3%
52	1	3	3	1	1	3	3	1	1	84%	3	1.2%
53	1	3	3	1	3	1	1	3	1	84%	3	2.6%
54	1	3	3	1	3	1	3	3	3	90%	3	1.9%
55	1	3	3	1	3	3	1	3	3	90%	3	0.4%
56	1	3	3	1	3	3	3	3	1	85%	3	2.5%
57	1	3	3	3	1	1	1	3	1	84%	3	1.1%
58	1	3	3	3	1	1	3	3	3	87%	3	0.1%
59	1	3	3	3	1	3	1	3	3	92%	3	0.4%
60	1	3	3	3	1	3	3	3	1	84%	3	1.0%
61	1	3	3	3	3	1	1	1	1	86%	3	0.9%
62	1	3	3	3	3	1	3	1	3	88%	3	2.1%
63	1	3	3	3	3	3	1	1	3	93%	3	0.6%
64	1	3	3	3	3	3	3	1	1	89%	3	0.7%
65	3	1	1	1	1	1	1	3	3	64%	3	7.0%

**66**	3	1	1	1	1	1	3	3	1	47%	3	1.0%
**67**	3	1	1	1	1	3	1	3	1	61%	3	2.6%
**68**	3	1	1	1	1	3	3	3	3	92%	3	0.6%
**69**	3	1	1	1	3	1	1	1	3	73%	3	4.5%
**70**	3	1	1	1	3	1	3	1	1	63%	3	2.1%
**71**	3	1	1	1	3	3	1	1	1	79%	3	3.3%
**72**	3	1	1	1	3	3	3	1	3	94%	3	0.7%
**73**	3	1	1	3	1	1	1	1	3	86%	3	1.9%
**74**	3	1	1	3	1	1	3	1	1	69%	3	1.0%
**75**	3	1	1	3	1	3	1	1	1	76%	3	1.9%
**76**	3	1	1	3	1	3	3	1	3	95%	3	1.1%
**77**	3	1	1	3	3	1	1	3	3	87%	3	1.2%
**78**	3	1	1	3	3	1	3	3	1	76%	3	1.3%
**79**	3	1	1	3	3	3	1	3	1	84%	3	1.4%
**80**	3	1	1	3	3	3	3	3	3	97%	3	0.3%
**81**	3	1	3	1	1	1	1	1	1	71%	3	2.6%
**82**	3	1	3	1	1	1	3	1	3	89%	3	1.0%
**83**	3	1	3	1	1	3	1	1	3	99%	3	0.0%
**84**	3	1	3	1	1	3	3	1	1	89%	3	0.6%
**85**	3	1	3	1	3	1	1	3	1	67%	3	1.6%
**86**	3	1	3	1	3	1	3	3	3	84%	3	2.5%
**87**	3	1	3	1	3	3	1	3	3	98%	3	0.2%
**88**	3	1	3	1	3	3	3	3	1	87%	3	1.1%
**89**	3	1	3	3	1	1	1	3	1	85%	3	1.3%
**90**	3	1	3	3	1	1	3	3	3	91%	3	0.5%
**91**	3	1	3	3	1	3	1	3	3	99%	3	0.1%
**92**	3	1	3	3	1	3	3	3	1	93%	3	0.3%
**93**	3	1	3	3	3	1	1	1	1	82%	3	1.1%
**94**	3	1	3	3	3	1	3	1	3	94%	3	0.6%
**95**	3	1	3	3	3	3	1	1	3	99%	3	0.1%
**96**	3	1	3	3	3	3	3	1	1	94%	3	0.4%
**97**	3	3	1	1	1	1	1	1	1	59%	3	4.0%
**98**	3	3	1	1	1	1	3	1	3	87%	3	0.9%
**99**	3	3	1	1	1	3	1	1	3	88%	3	1.3%
**100**	3	3	1	1	1	3	3	1	1	81%	3	2.4%
**101**	3	3	1	1	3	1	1	3	1	74%	3	0.9%
**102**	3	3	1	1	3	1	3	3	3	89%	3	1.2%
**103**	3	3	1	1	3	3	1	3	3	87%	3	0.6%
**104**	3	3	1	1	3	3	3	3	1	85%	3	0.5%
**105**	3	3	1	3	1	1	1	3	1	71%	3	1.3%
**106**	3	3	1	3	1	1	3	3	3	91%	3	0.9%
**107**	3	3	1	3	1	3	1	3	3	90%	3	0.5%
**108**	3	3	1	3	1	3	3	3	1	82%	3	1.3%
**109**	3	3	1	3	3	1	1	1	1	80%	3	1.6%
**110**	3	3	1	3	3	1	3	1	3	89%	3	2.1%
**111**	3	3	1	3	3	3	1	1	3	91%	3	1.3%
**112**	3	3	1	3	3	3	3	1	1	86%	3	1.4%
**113**	3	3	3	1	1	1	1	3	3	92%	3	0.2%
**114**	3	3	3	1	1	1	3	3	1	78%	3	1.1%
**115**	3	3	3	1	1	3	1	3	1	85%	3	1.0%
**116**	3	3	3	1	1	3	3	3	3	89%	3	1.3%
**117**	3	3	3	1	3	1	1	1	3	90%	3	1.3%
**118**	3	3	3	1	3	1	3	1	1	83%	3	2.7%
**119**	3	3	3	1	3	3	1	1	1	90%	3	0.4%
**120**	3	3	3	1	3	3	3	1	3	88%	3	0.6%
**121**	3	3	3	3	1	1	1	1	3	90%	3	0.7%
**122**	3	3	3	3	1	1	3	1	1	85%	3	1.1%
**123**	3	3	3	3	1	3	1	1	1	91%	3	0.6%
**124**	3	3	3	3	1	3	3	1	3	90%	3	0.9%
**125**	3	3	3	3	3	1	1	3	3	93%	3	0.4%
**126**	3	3	3	3	3	1	3	3	1	85%	3	1.6%
**127**	3	3	3	3	3	3	1	3	1	89%	3	0.3%
**128**	3	3	3	3	3	3	3	3	3	91%	3	0.4%
**129**	1	1	1	1	1	1	1	1	1	2%	3	7.8%
**130**	1	1	2	2	1	3	2	3	3	96%	3	0.4%
**131**	1	1	3	3	1	2	3	2	2	91%	3	0.2%
**132**	1	2	1	2	3	2	1	2	3	90%	3	0.8%
**133**	1	2	2	3	3	1	2	1	2	80%	3	2.6%
**134**	1	2	3	1	3	3	3	3	1	87%	3	0.6%
**135**	1	3	1	3	2	3	1	3	2	75%	3	1.9%
**136**	1	3	2	1	2	2	2	2	1	56%	3	2.8%
**137**	1	3	3	2	2	1	3	1	3	87%	3	1.0%
**138**	2	1	1	2	2	2	2	1	2	39%	3	4.1%
**139**	2	1	2	3	2	1	3	3	1	68%	3	1.7%
**140**	2	1	3	1	2	3	1	2	3	98%	3	0.1%

**141**	2	2	1	3	1	3	2	2	1	69%	3	2.8%
**142**	2	2	2	1	1	2	3	1	3	90%	3	0.4%
**143**	2	2	3	2	1	1	1	3	2	88%	3	1.0%
**144**	2	3	1	1	3	1	2	3	3	85%	3	1.3%
**145**	2	3	2	2	3	3	3	2	2	86%	3	1.1%
**146**	2	3	3	3	3	2	1	1	1	87%	3	0.6%
**147**	3	1	1	3	3	3	3	1	3	96%	3	0.1%
**148**	3	1	2	1	3	2	1	3	2	84%	3	0.3%
**149**	3	1	3	2	3	1	2	2	1	58%	3	6.4%
**150**	3	2	1	1	2	1	3	2	2	71%	3	2.9%
**151**	3	2	2	2	2	3	1	1	1	84%	3	1.8%
**152**	3	2	3	3	2	2	2	3	3	93%	3	0.4%
**153**	3	3	1	2	1	2	3	3	1	75%	3	3.0%
**154**	3	3	2	3	1	1	1	2	3	91%	3	0.8%
**155**	3	3	3	1	1	3	2	1	2	86%	3	0.6%
**SR**										70%	3	1.8%

Drug dose “1”, “2” and “3” represent 0%, 7.5% and 15% of the drug effect level, respectively. Data shown are mean % inhibition, number of replicates (N), and standard error (SE). SR, Standard Regimen tested at 15% of the drug effect level.
