# Supplementary material for: Artificial intelligence enabled parabolic response surface platform identifies ultra-rapid near-universal TB drug treatment regimens comprising approved drugs
Source: PLoS One. 2019 May 10;14(5):e0215607. doi: 10.1371/journal.pone.0215607 (PMC6510528; doi:10.1371/journal.pone.0215607)
Supplement: S5 Table — (PDF) [file pone.0215607.s005.pdf]

S5 Table. Iteration 3A, five-level orthogonal array central composite design and experimental results.

Run/Drug	AC	CFZ	PA824	RIF	BDQ	DLM	% Inhibition Mean	N	SE
Control 1 (no infection control)							97%	4	0.3%
Control 2 (no IPTG control)							98%	4	0.2%
Control 3 (no drug control)							0%	34	1.8%
Control 4 (all drug control)							87%	2	1.3%
1	1	1	1	1	1	1	0%	3	9.9%
2	1	1	1	1	5	5	50%	3	3.8%
3	1	1	1	5	1	5	86%	3	1.4%
4	1	1	1	5	5	1	31%	3	7.3%
5	1	1	5	1	1	5	81%	3	1.7%
6	1	1	5	1	5	1	51%	3	3.9%
7	1	1	5	5	1	1	59%	3	3.1%
8	1	1	5	5	5	5	94%	3	0.2%
9	1	5	1	1	1	5	62%	3	4.3%
10	1	5	1	1	5	1	32%	3	8.0%
11	1	5	1	5	1	1	20%	3	4.9%
12	1	5	1	5	5	5	54%	3	6.6%
13	1	5	5	1	1	1	48%	3	2.7%
14	1	5	5	1	5	5	79%	3	0.8%
15	1	5	5	5	1	5	85%	3	1.5%
16	1	5	5	5	5	1	50%	3	3.3%
17	5	1	1	1	1	5	58%	3	3.5%
18	5	1	1	1	5	1	18%	3	10.7%
19	5	1	1	5	1	1	53%	3	7.5%
20	5	1	1	5	5	5	90%	3	0.0%
21	5	1	5	1	1	1	67%	3	2.4%
22	5	1	5	1	5	5	85%	3	2.5%
23	5	1	5	5	1	5	95%	3	0.7%
24	5	1	5	5	5	1	70%	3	1.0%
25	5	5	1	1	1	1	38%	3	4.0%
26	5	5	1	1	5	5	68%	3	3.0%
27	5	5	1	5	1	5	77%	3	1.2%
28	5	5	1	5	5	1	41%	3	6.9%
29	5	5	5	1	1	5	90%	3	0.9%
30	5	5	5	1	5	1	64%	3	4.3%
31	5	5	5	5	1	1	58%	3	3.3%
32	5	5	5	5	5	5	87%	3	0.8%
33	1	1	1	1	1	1	15%	3	5.4%
34	1	3	3	3	3	3	46%	3	1.4%
35	1	5	5	5	5	5	83%	3	0.5%
36	3	1	1	3	3	5	81%	3	1.4%
37	3	3	3	5	5	1	32%	3	1.4%
38	3	5	5	1	1	3	75%	3	2.1%
39	5	1	3	1	5	3	61%	3	0.7%
40	5	3	5	3	1	5	91%	3	0.4%
41	5	5	1	5	3	1	36%	3	2.4%
42	1	1	5	5	3	3	83%	3	0.6%
43	1	3	1	1	5	5	63%	3	4.9%

**44**	1	5	3	3	1	1	24%	3	5.4%
**45**	3	1	3	5	1	5	92%	3	0.2%
**46**	3	3	5	1	3	1	66%	3	2.9%
**47**	3	5	1	3	5	3	34%	3	5.9%
**48**	5	1	5	3	5	1	70%	3	1.7%
**49**	5	3	1	5	1	3	39%	3	4.7%
**50**	5	5	3	1	3	5	82%	3	0.3%
**51**	3	3	3	3	3	3	53%	3	3.7%
**52**	3	5	5	4	2	1	53%	3	1.2%
**53**	3	4	4	1	5	2	55%	3	5.5%
**54**	3	2	2	5	1	4	78%	3	2.4%
**55**	3	1	1	2	4	5	75%	3	0.8%
**56**	5	3	5	5	5	5	90%	3	1.4%
**57**	5	5	4	2	1	3	59%	3	1.5%
**58**	5	4	2	3	4	1	34%	3	3.7%
**59**	5	2	1	4	3	2	34%	3	6.7%
**60**	5	1	3	1	2	4	77%	3	2.5%
**61**	4	3	4	4	4	4	83%	3	1.9%
**62**	4	5	2	1	3	5	77%	3	1.0%
**63**	4	4	1	5	2	3	13%	3	7.4%
**64**	4	2	3	2	5	1	45%	3	4.3%
**65**	4	1	5	3	1	2	78%	3	0.3%
**66**	2	3	2	2	2	2	13%	3	1.6%
**67**	2	5	1	3	5	4	39%	3	2.6%
**68**	2	4	3	4	1	5	81%	3	1.6%
**69**	2	2	5	1	4	3	55%	3	2.3%
**70**	2	1	4	5	3	1	54%	3	3.5%
**71**	1	3	1	1	1	1	3%	3	7.1%
**72**	1	5	3	5	4	2	31%	3	1.5%
**73**	1	4	5	2	3	4	77%	3	1.0%
**74**	1	2	4	3	2	5	88%	3	0.4%
**75**	1	1	2	4	5	3	26%	3	5.2%
**SR**							95%	3	0.2%

Drug dose “1”, “2”, “3”, “4” and “5” represent 0%, 5%, 10%, 15% and 20% of the drug effect level, respectively. Data shown are mean % inhibition, number of replicates (N), and standard error (SE). SR, Standard Regimen tested at 20% of the drug effect level.
