# Supplementary material for: Artificial intelligence enabled parabolic response surface platform identifies ultra-rapid near-universal TB drug treatment regimens comprising approved drugs
Source: PLoS One. 2019 May 10;14(5):e0215607. doi: 10.1371/journal.pone.0215607 (PMC6510528; doi:10.1371/journal.pone.0215607)
Supplement: S6 Table — (PDF) [file pone.0215607.s006.pdf]

S6 Table. Iteration 3B, five-level orthogonal array central composite design and experimental results.

							% Inhibition		
							Mean	N	SE
Control 1 (no infection control)							100%	4	0.1%
Control 2 (no IPTG control)							100%	4	0.1%
Control 3 (no drug control)							0%	27	1.0%
Control 4 (all drug control)							95%	2	0.3%
Run/Drug	AC	CFZ	SQ109	RIF	BDQ	DLM			
1	1	1	1	1	1	1	-8%	3	1.7%
2	1	1	1	1	5	5	21%	3	0.9%
3	1	1	1	5	1	5	68%	3	2.5%
4	1	1	1	5	5	1	18%	3	3.0%
5	1	1	5	1	1	5	36%	3	1.9%
6	1	1	5	1	5	1	32%	3	2.9%
7	1	1	5	5	1	1	66%	3	3.3%
8	1	1	5	5	5	5	85%	3	2.4%
9	1	5	1	1	1	5	56%	3	1.9%
10	1	5	1	1	5	1	51%	3	0.8%
11	1	5	1	5	1	1	34%	3	2.5%
12	1	5	1	5	5	5	58%	3	4.0%
13	1	5	5	1	1	1	63%	3	3.3%
14	1	5	5	1	5	5	81%	3	0.5%
15	1	5	5	5	1	5	83%	3	3.2%
16	1	5	5	5	5	1	83%	3	2.2%
17	5	1	1	1	1	5	39%	3	5.5%
18	5	1	1	1	5	1	9%	3	5.3%
19	5	1	1	5	1	1	48%	3	2.1%
20	5	1	1	5	5	5	86%	3	0.3%
21	5	1	5	1	1	1	38%	3	3.2%
22	5	1	5	1	5	5	63%	3	2.8%
23	5	1	5	5	1	5	94%	3	0.6%
24	5	1	5	5	5	1	81%	3	0.6%
25	5	5	1	1	1	1	47%	3	1.1%
26	5	5	1	1	5	5	70%	3	2.2%
27	5	5	1	5	1	5	67%	3	2.4%
28	5	5	1	5	5	1	52%	3	1.9%
29	5	5	5	1	1	5	89%	3	0.3%
30	5	5	5	1	5	1	76%	3	1.0%
31	5	5	5	5	1	1	87%	3	2.4%
32	5	5	5	5	5	5	95%	3	0.8%
33	1	1	1	1	1	1	-15%	3	3.6%
34	1	3	3	3	3	3	53%	3	4.4%
35	1	5	5	5	5	5	93%	3	0.5%
36	3	1	1	3	3	5	90%	3	1.5%
37	3	3	3	5	5	1	64%	3	1.9%
38	3	5	5	1	1	3	81%	3	2.2%
39	5	1	3	1	5	3	36%	3	2.4%
40	5	3	5	3	1	5	90%	3	1.1%
41	5	5	1	5	3	1	55%	3	8.2%
42	1	1	5	5	3	3	50%	3	5.5%
43	1	3	1	1	5	5	62%	3	3.0%

**44**	1	5	3	3	1	1	58%	3	3.4%
**45**	3	1	3	5	1	5	94%	3	0.3%
**46**	3	3	5	1	3	1	61%	3	2.4%
**47**	3	5	1	3	5	3	47%	3	5.2%
**48**	5	1	5	3	5	1	77%	3	2.0%
**49**	5	3	1	5	1	3	54%	3	7.8%
**50**	5	5	3	1	3	5	86%	3	0.6%
**51**	3	3	3	3	3	3	56%	3	0.5%
**52**	3	5	5	4	2	1	70%	3	3.3%
**53**	3	4	4	1	5	2	71%	3	2.0%
**54**	3	2	2	5	1	4	75%	3	2.8%
**55**	3	1	1	2	4	5	84%	3	0.8%
**56**	5	3	5	5	5	5	95%	3	0.6%
**57**	5	5	4	2	1	3	73%	3	0.6%
**58**	5	4	2	3	4	1	49%	3	3.0%
**59**	5	2	1	4	3	2	44%	3	1.5%
**60**	5	1	3	1	2	4	57%	3	2.0%
**61**	4	3	4	4	4	4	87%	3	2.2%
**62**	4	5	2	1	3	5	83%	3	0.3%
**63**	4	4	1	5	2	3	50%	3	3.8%
**64**	4	2	3	2	5	1	62%	3	5.2%
**65**	4	1	5	3	1	2	80%	3	2.6%
**66**	2	3	2	2	2	2	20%	3	5.9%
**67**	2	5	1	3	5	4	58%	3	2.1%
**68**	2	4	3	4	1	5	86%	3	0.8%
**69**	2	2	5	1	4	3	68%	3	3.2%
**70**	2	1	4	5	3	1	78%	3	1.5%
**71**	1	3	1	1	1	1	33%	3	4.9%
**72**	1	5	3	5	4	2	70%	3	2.5%
**73**	1	4	5	2	3	4	79%	3	2.2%
**74**	1	2	4	3	2	5	85%	3	1.2%
**75**	1	1	2	4	5	3	39%	3	2.9%
**SR**							99%	3	0.1%

Drug dose “1”, “2”, “3”, “4” and “5” represent 0%, 5%, 10%, 15% and 20% of the drug effect level, respectively. Data shown are mean % inhibition, number of replicates (N), and standard error (SE). SR, Standard Regimen tested at 20% of the drug effect level.
