# Supplementary material for: Artificial intelligence enabled parabolic response surface platform identifies ultra-rapid near-universal TB drug treatment regimens comprising approved drugs
Source: PLoS One. 2019 May 10;14(5):e0215607. doi: 10.1371/journal.pone.0215607 (PMC6510528; doi:10.1371/journal.pone.0215607)
Supplement: S7 Table — (PDF) [file pone.0215607.s007.pdf]

S7 Table. Iteration 3C, three-level orthogonal array central composite design and experimental results.

								% Inhibition		
								Mean	N	SE
Control 1 (no infection control)								95%	4	0.4%
Control 2 (no IPTG control)								92%	4	0.4%
Control 3 (no drug control)								0%	26	1.2%
Control 4 (all drug control, at 7.5% drug effect level)								42%	2	2.7%
Run/Drug	AC	CFZ	PA824	RIF	SQ109	BDQ	DLM			
1	1	1	1	1	1	1	3	19%	3	3.8%
2	1	1	1	1	1	3	1	33%	3	2.0%
3	1	1	1	1	3	1	1	25%	3	3.9%
4	1	1	1	1	3	3	3	54%	3	1.3%
5	1	1	1	3	1	1	1	26%	3	4.8%
6	1	1	1	3	1	3	3	61%	3	2.6%
7	1	1	1	3	3	1	3	77%	3	1.9%
8	1	1	1	3	3	3	1	63%	3	1.5%
9	1	1	3	1	1	1	1	21%	3	2.3%
10	1	1	3	1	1	3	3	54%	3	4.3%
11	1	1	3	1	3	1	3	65%	3	1.6%
12	1	1	3	1	3	3	1	69%	3	1.4%
13	1	1	3	3	1	1	3	71%	3	2.9%
14	1	1	3	3	1	3	1	43%	3	1.8%
15	1	1	3	3	3	1	1	80%	3	1.3%
16	1	1	3	3	3	3	3	77%	3	1.3%
17	1	3	1	1	1	1	1	11%	3	6.5%
18	1	3	1	1	1	3	3	51%	3	3.4%
19	1	3	1	1	3	1	3	51%	3	2.1%
20	1	3	1	1	3	3	1	62%	3	4.1%
21	1	3	1	3	1	1	3	54%	3	3.6%
22	1	3	1	3	1	3	1	33%	3	3.3%
23	1	3	1	3	3	1	1	68%	3	0.8%
24	1	3	1	3	3	3	3	78%	3	0.2%
25	1	3	3	1	1	1	3	49%	3	2.9%
26	1	3	3	1	1	3	1	52%	3	2.0%
27	1	3	3	1	3	1	1	45%	3	4.6%
28	1	3	3	1	3	3	3	79%	3	0.6%
29	1	3	3	3	1	1	1	37%	3	3.1%
30	1	3	3	3	1	3	3	66%	3	1.1%
31	1	3	3	3	3	1	3	79%	3	0.5%
32	1	3	3	3	3	3	1	77%	3	1.5%
33	3	1	1	1	1	1	1	25%	3	4.8%
34	3	1	1	1	1	3	3	57%	3	2.1%
35	3	1	1	1	3	1	3	64%	3	1.4%
36	3	1	1	1	3	3	1	78%	3	1.1%
37	3	1	1	3	1	1	3	77%	3	1.6%
38	3	1	1	3	1	3	1	56%	3	1.0%
39	3	1	1	3	3	1	1	84%	3	0.8%
40	3	1	1	3	3	3	3	83%	3	0.3%
41	3	1	3	1	1	1	3	59%	3	2.0%
42	3	1	3	1	1	3	1	56%	3	3.0%
43	3	1	3	1	3	1	1	62%	3	1.0%

**44**	3	1	3	1	3	3	3	76%	3	1.5%
**45**	3	1	3	3	1	1	1	55%	3	2.6%
**46**	3	1	3	3	1	3	3	77%	3	1.0%
**47**	3	1	3	3	3	1	3	84%	3	1.2%
**48**	3	1	3	3	3	3	1	72%	3	1.0%
**49**	3	3	1	1	1	1	3	56%	3	2.8%
**50**	3	3	1	1	1	3	1	49%	3	1.6%
**51**	3	3	1	1	3	1	1	51%	3	1.7%
**52**	3	3	1	1	3	3	3	68%	3	1.6%
**53**	3	3	1	3	1	1	1	38%	3	2.7%
**54**	3	3	1	3	1	3	3	62%	3	0.6%
**55**	3	3	1	3	3	1	3	75%	3	0.3%
**56**	3	3	1	3	3	3	1	69%	3	1.0%
**57**	3	3	3	1	1	1	1	44%	3	3.9%
**58**	3	3	3	1	1	3	3	65%	3	1.2%
**59**	3	3	3	1	3	1	3	68%	3	1.2%
**60**	3	3	3	1	3	3	1	70%	3	0.7%
**61**	3	3	3	3	1	1	3	76%	3	0.9%
**62**	3	3	3	3	1	3	1	45%	3	4.3%
**63**	3	3	3	3	3	1	1	74%	3	1.3%
**64**	3	3	3	3	3	3	3	74%	3	1.1%
**65**	1	1	1	1	1	1	1	6%	3	8.0%
**66**	1	2	2	2	2	2	2	36%	3	2.1%
**67**	1	3	3	3	3	3	3	70%	3	1.9%
**68**	2	1	1	2	2	3	3	48%	3	2.8%
**69**	2	2	2	3	3	1	1	66%	3	0.2%
**70**	2	3	3	1	1	2	2	49%	3	1.5%
**71**	3	1	2	1	3	2	3	66%	3	1.2%
**72**	3	2	3	2	1	3	1	46%	3	3.1%
**73**	3	3	1	3	2	1	2	58%	3	0.7%
**74**	1	1	3	3	2	2	1	37%	3	3.0%
**75**	1	2	1	1	3	3	2	58%	3	3.5%
**76**	1	3	2	2	1	1	3	50%	3	1.3%
**77**	2	1	2	3	1	3	2	54%	3	1.8%
**78**	2	2	3	1	2	1	3	61%	3	1.3%
**79**	2	3	1	2	3	2	1	41%	3	1.8%
**80**	3	1	3	2	3	1	2	67%	3	0.7%
**81**	3	2	1	3	1	2	3	76%	3	1.0%
**82**	3	3	2	1	2	3	1	56%	3	1.4%
**SR (H)**								91%	3	0.6%
**SR (M)**								57%	3	0.5%

Drug dose “1”, “2” and “3” represent 0%, 7.5% and 15% of the drug effect level, respectively. Data shown are mean % inhibition, number of replicates (N), and standard error (SE). SR (H), Standard Regimen tested at 15% of the drug effect level. SR (M), Standard Regimen tested at 7.5% of the drug effect level.
