# Supplementary material for: Artificial intelligence enabled parabolic response surface platform identifies ultra-rapid near-universal TB drug treatment regimens comprising approved drugs
Source: PLoS One. 2019 May 10;14(5):e0215607. doi: 10.1371/journal.pone.0215607 (PMC6510528; doi:10.1371/journal.pone.0215607)
Supplement: S8 Table — (PDF) [file pone.0215607.s008.pdf]

S8 Table. *In vivo* treatment efficacy of the 14 top 4-drug experimental regimens identified from studies in *M. tuberculosis* infected THP-1 macrophages using the PRS platform.

Group	Treatment[a]	Lung burden in mouse (log_{10} CFU)					Mean ± SE
	Drug combination (mg/kg)	1	2	3	4	5	
A	Sham						
		7.59	7.33	7.61	7.63	7.65	7.56 ± 0.06
B	Standard Regimen[b]						
	INH,RIF,EMB,PZA (25,10,100,150)	4.69	4.78	4.78	4.79	4.36	4.68 ± 0.08
C	PRS Regimen II						
	CFZ,BDQ,PZA,EMB (25,30,450,100)	0.78	0.78	0.00	0.95	0.60	0.62 ± 0.17
D	Experimental Regimen[c]						
	RPT,AC,DLM,SQ109 (10,200-50,2.5,25)	3.78	3.61	3.77	3.83	3.79	3.76 ± 0.04
E	Experimental Regimen						
	RPT,AC,DLM,PA824 (10,200-50,2.5,100)	≥5.00	≥5.00	≥5.00	≥5.00	≥5.00	
F	Experimental Regimen						
	RPT,AC,PA824,SQ109 (10,200-50,100,25)	≥5.00	≥5.00	≥5.00	≥5.00	≥5.00	
G	Experimental Regimen						
	RPT,AC,BDQ,SQ109 (10,200-50,30,25)	3.06	3.01	2.60	2.76	2.89	2.86 ± 0.08
H	Experimental Regimen						
	RPT,AC,CFZ,DLM (10,200-50,25,2.5)	2.65	3.33	3.31	2.99	3.28	3.11 ± 0.13
I	Experimental Regimen						
	RPT,AC,BDQ,DLM (10,200-50,30,2.5)	2.95	3.17	2.77	2.64	3.20	2.95 ± 0.11
J	Experimental Regimen						
	RPT,AC,BDQ,CFZ (10,200-50,30,25)	1.56	2.10	2.27	1.87	1.74	1.91 ± 0.13
K	Experimental Regimen						
	RPT,DLM,PA824,SQ109 (10,2.5,100,25)	≥5.00	≥5.00	≥5.00	≥5.00	≥5.00	
L	Experimental Regimen						
	CFZ,AC,BDQ,DLM (25,200-50,30,2.5)	1.40	1.72	1.20	1.78	1.91	1.60 ± 0.13
M	Experimental Regimen						
	CFZ,AC,BDQ,PZA (25,200-50,30,450)	0.48	1.08	0.60	0.85	0.30	0.66 ± 0.14
N	Experimental Regimen						
	CFZ,AC,DLM,PA824 (25,200-50,2.5,100)	≥5.00	≥5.00	≥5.00	≥5.00	≥5.00	
O	Experimental Regimen						
	CFZ,BDQ,PA824,SQ109 (25,30,100,25)	3.24	3.15	3.40	3.28	3.24	3.26 ± 0.04
P	Experimental Regimen						
	CFZ,BDQ,DLM,PZA (25,30,2.5,450)	0.74	1.26	1.23	0.70	0.90	0.97 ± 0.12
Q	Experimental Regimen						
	CFZ,BDQ,DLM,PA824 (25,30,2.5,100)	3.34	3.12	3.23	3.19	3.48	3.27 ± 0.06

[a]BALB/c mice (17 groups, 5 mice/group) were infected with 2.5 log_{10} CFU of *M. tuberculosis* Erdman by aerosol. Two weeks later, the number of bacteria in the lung increased to 6.01 logs at which time the treatment were initiated. The mice received sham treatment or treatment with the Standard Regimen, PRS Regimen II, or one of the fourteen top 4-drug experimental regimens by oral gavage 5 days per week (Monday-Friday) for 3 weeks. Three days after the last treatment, mice were euthanized to determine the lung burden of *M. tuberculosis*.

[b]RIF in the Standard Regimen represents rifampicin.

[c]RPT in all experimental regimens represents rifapentine.
