# Supplementary material for: Artificial intelligence enabled parabolic response surface platform identifies ultra-rapid near-universal TB drug treatment regimens comprising approved drugs
Source: PLoS One. 2019 May 10;14(5):e0215607. doi: 10.1371/journal.pone.0215607 (PMC6510528; doi:10.1371/journal.pone.0215607)
Supplement: S9 Table — (PDF) [file pone.0215607.s009.pdf]

**S9 Table. Mouse lung burden of *M. tuberculosis* in PRS Regimens IV and V optimal dose finding study.**

| Treatment[a] | Dose | Concentration (mg/kg) | $Log_{10}$ CFU (Mean ± SE) | | |
|---|---|---|---|---|---|
| | | | Day 1 | Day 14 | Day 35 |
| Sham | | | 2.24 ± 0.03 | 6.17 ± 0.05 | 7.18 ± 0.10 |
| Standard Regimen | | | | | |
| INH, RIF, EMB, PZA | | 25, 10, 100, 150 | | | 4.05 ± 0.17 |
| PRS Regimen II | | | | | |
| CFZ, BDQ, PZA, EMB | | 25, 30, 450, 100 | | | 1.16 ± 0.13 |
| PRS Regimen IV | | | | | |
| CFZ, BDQ, PZA, AC | HLHL | 25, 5.6, 450, 66.7-16.7 | | | 1.98 ± 0.12 |
| | HHLL | 25, 50, 50, 66.7-16.7 | | | 0.55 ± 0.19 |
| | HLLH | 25, 5.6, 50, 600-150 | | | 2.74 ± 0.13 |
| | HHHH | 25, 50, 450, 600-150 | | | 1.01 ± 0.26 |
| | HHLH | 25, 50, 50, 600-150 | | | 0.77 ± 0.14 |
| | HLHH | 25, 5.6, 450, 600-150 | | | 1.87 ± 0.18 |
| | HHHL | 25, 50, 450, 66.7-16.7 | | | 1.13 ± 0.13 |
| | HMMH | 25, 16.7, 150, 600-150 | | | 1.22 ± 0.08 |
| | HHMM | 25, 50, 150, 200-50 | | | 1.03 ± 0.13 |
| | HMHM | 25, 16.7, 450, 200-50 | | | 1.00 ± 0.06 |
| PRS Regimen V | | | | | |
| CFZ, BDQ, PZA, DLM | HLHL | 25, 5.6, 450, 0.83 | | | 2.13 ± 0.05 |
| | HHLL | 25, 50, 50, 0.83 | | | 0.96 ± 0.11 |
| | HLLH | 25, 5.6, 50, 7.5 | | | 3.06 ± 0.09 |
| | HHHH | 25, 50, 450, 7.5 | | | 1.06 ± 0.19 |
| | HHLH | 25, 50, 50, 7.5 | | | 1.14 ± 0.06 |
| | HLHH | 25, 5.6, 450, 7.5 | | | 2.22 ± 0.18 |
| | HHHL | 25, 50, 450, 0.83 | | | 0.97 ± 0.14 |
| | HMMH | 25, 16.7, 150, 7.5 | | | 1.60 ± 0.09 |
| | HHMM | 25, 50, 150, 2.5 | | | 0.73 ± 0.11 |
| | HMHM | 25, 16.7, 450, 2.5 | | | 1.32 ± 0.16 |

[a]BALB/c mice were infected by aerosol with *M. tuberculosis* Erdman at Day 0. Two mice were euthanized one day later (Day 1) to determine the number of bacteria delivered to the lung. Three mice were euthanized two weeks later (Day 14) to determine the lung burden of bacteria at the start of treatment. Mice (5 per group) were sham treated or treated with the Standard Regimen, PRS Regimen II or a dose variation of PRS Regimens IV and V by oral gavage, 5 days per week (Monday-Friday) for 3 weeks. Mice were euthanized three days after the last treatment to determine *M. tuberculosis* burdens in the lung.
