# Supplementary material for: Artificial intelligence enabled parabolic response surface platform identifies ultra-rapid near-universal TB drug treatment regimens comprising approved drugs
Source: PLoS One. 2019 May 10;14(5):e0215607. doi: 10.1371/journal.pone.0215607 (PMC6510528; doi:10.1371/journal.pone.0215607)
Supplement: S10 Table — (PDF) [file pone.0215607.s010.pdf]

S10 Table. Mouse lung burden of *M. tuberculosis* in treatment efficacy study.

Treatment[a]	Drug combination (dose in mg/kg)	Log_{10} CFU (Mean ± SE)				
		Week 0	Week 3[b]	Week 4[c]	Week 5[d]	Week 6[e]
Sham		6.25 ± 0.06	6.94 ± 0.09	6.83 ± 0.03	6.80 ± 0.09	6.75 ± 0.07
Standard Regimen	INH,RIF,EMB,PZA (25,10,100,150)		4.52 ± 0.07	3.75 ± 0.09	2.88 ± 0.10	1.99 ± 0.10
PRS Regimen III	CFZ,BDQ,PZA,SQ109 (25,30,450,25)		0.51 ± 0.10	0.00 ± 0.00	0.00 ± 0.00	0.00 ± 0.00
PRS Regimen IV	CFZ,BDQ,PZA,AC (25,37,50,66.7-16.7)		0.47 ± 0.13	0.00 ± 0.00	0.00 ± 0.00	0.00 ± 0.00
PRS Regimen V	CFZ,BDQ,PZA,DLM (25,40,185,0.83)		0.36 ± 0.15	0.00 ± 0.00	0.00 ± 0.00	0.00 ± 0.00
PRS Regimen VI	CFZ,BDQ,PZA (25,40,185)		0.28 ± 0.17	0.00 ± 0.00	0.00 ± 0.00	0.00 ± 0.00

[a]BALB/c mice were infected by aerosol with *M. tuberculosis* Erdman. Six mice were euthanized two weeks later (Week 0) to determine the lung burden of bacteria at the start of treatment. Mice (5 per group) were sham treated or treated with the Standard Regimen or one of the PRS Regimens (III-VI) by oral gavage, 5 days per week (Monday-Friday) for 3, 4, 5, and 6 weeks. Mice were euthanized three days after the last treatment to determine *M. tuberculosis* burdens in the lung.

[b]One mouse in the group treated with PRS Regimen V had no CFU detected in the entire lung for which a CFU of 1 was used for calculation of the log_{10}CFU mean and standard error for the group.

[c]All of the mice in groups treated with PRS Regimens III and V were culture negative for the entire lung. For mice treated with PRS Regimens IV and VI, each group had three mice with no detectable *M. tuberculosis* CFU in the entire lung. For mice with no bacterial CFU in the lung, a CFU of 1 was used for calculation of the log_{10}CFU mean and standard error for the group.

[d]All of the mice in groups treated with PRS Regimens (III-VI) were culture negative for the entire lung. For those mice, a CFU of 1 was used for calculation of the log_{10}CFU mean and standard error for the group.

[e]All of the mice in groups treated with PRS Regimens (III-VI) were culture negative for the entire lung. For those mice, a CFU of 1 was used for calculation of the log_{10}CFU mean and standard error for the group.
