# Supplementary material for: Artificial intelligence enabled parabolic response surface platform identifies ultra-rapid near-universal TB drug treatment regimens comprising approved drugs
Source: PLoS One. 2019 May 10;14(5):e0215607. doi: 10.1371/journal.pone.0215607 (PMC6510528; doi:10.1371/journal.pone.0215607)
Supplement: S11 Table — (PDF) [file pone.0215607.s011.pdf]

S11 Table. Mouse lung burden of *M. tuberculosis* in relapse study.

(A) Total lung CFU in mice 3 months after completion of 6 weeks treatment with the Standard Regimen comprising INH, RIF, EMB and PZA.

Mouse	Total lung CFU
	6 weeks
1	≥40,000
2	646
3	16,000
4	678
5	832
6	32,000
7	1,047
8	822
9	887
10	1,014

(B) Total lung CFU in mice 3 months after completion of 3, 4, and 5 weeks treatment with PRS Regimen III comprising CFZ, BDQ, PZA and SQ109 at 25, 30, 450 and 25 mg/kg.

Mouse	Total lung CFU		
	3 weeks	4 weeks	5 weeks
1	0	0	0
2	0	0	0
3	0	0	0
4	0	0	0
5	0	0	0
6	0	0	0
7	0	0	0
8	0	0	0
9	0	0	0
10	0	0	0

(C) Total lung CFU in mice 3 months after completion of 3, 4, 5, and 6 weeks treatment with PRS Regimen IV comprising CFZ, BDQ, PZA and AC at 25, 37, 50 and 66.7-16.7 mg/kg.

Mouse	Total lung CFU			
	3 weeks	4 weeks	5 weeks	6 weeks
1	0	1	0	0
2	97	0	0	0
3	0	0	contaminated	0
4	0	0	0	0
5	contaminated	0	0	0
6	10	0	0	0
7	0	0	0	0
8	0	0	0	0
9	0	0	0	0
10	0	0	0	0

(D) Total lung CFU in mice 3 months after completion of 3, 4, 5, and 6 weeks treatment with PRS Regimen V comprising CFZ, BDQ, PZA and DLM at 25, 40, 185 and 0.83 mg/kg.

Mouse	Total lung CFU			
	3 weeks	4 weeks	5 weeks	6 weeks
1	0	0	0	0
2	0	0	0	0
3	0	0	0	0
4	0	0	0	0
5	0	0	0	0
6	0	0	0	0
7	0	0	0	0
8	0	0		0
9	0	0		0
10	0	0		0

(E) Total lung CFU in mice 3 months after completion of 3, 4, 5, and 6 weeks treatment with PRS Regimen VI comprising CFZ, BDQ and PZA at 25, 40 and 185 mg/kg

Mouse	Total lung CFU			
	3 weeks	4 weeks	5 weeks	6 weeks
1	0	0	0	0
2	0	0	0	0
3	0	0	0	0
4	0	0	0	0
5	0	0	0	0
6	0	0	0	0
7	0	0	0	0
8	0	0	0	0
9	0	0	0	0
10	0	0	0	0
