# Supplementary material for: Artificial intelligence enabled parabolic response surface platform identifies ultra-rapid near-universal TB drug treatment regimens comprising approved drugs
Source: PLoS One. 2019 May 10;14(5):e0215607. doi: 10.1371/journal.pone.0215607 (PMC6510528; doi:10.1371/journal.pone.0215607)
Supplement: S1 Fig — (PDF) [file pone.0215607.s012.pdf]

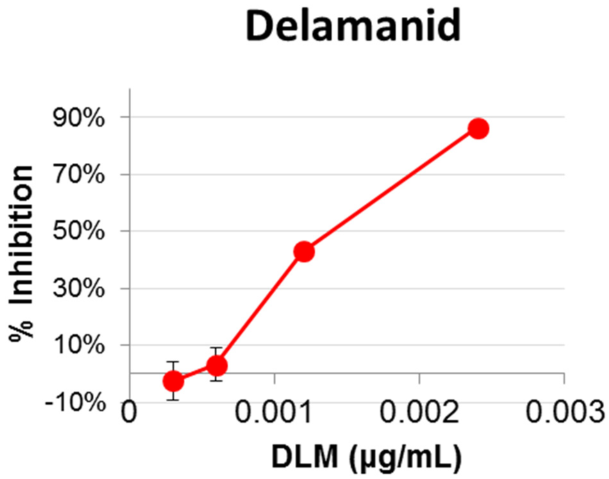

S1 Fig. Dose-response curve of delamanid (DLM). THP-1 monocytic cells were differentiated into macrophage-like cells with phorbol myristate acetate, infected with *Mycobacterium tuberculosis* expressing IPTG-inducible GFP (Mtb-iGFP) and treated with DLM in the presence of IPTG for 4 days. Images of the cultures after fixation and staining of the nuclei with Hoechst 33342 were acquired to quantitate the integrated GFP fluorescence intensity and the number of macrophage nuclei. Inhibition is calculated as shown in Equation 1 in the Materials and methods. Data shown are mean % inhibition ± standard error of 3 independent cultures.
