# Supplementary material for: Artificial intelligence enabled parabolic response surface platform identifies ultra-rapid near-universal TB drug treatment regimens comprising approved drugs
Source: PLoS One. 2019 May 10;14(5):e0215607. doi: 10.1371/journal.pone.0215607 (PMC6510528; doi:10.1371/journal.pone.0215607)
Supplement: S2 Fig — (PDF) [file pone.0215607.s013.pdf]

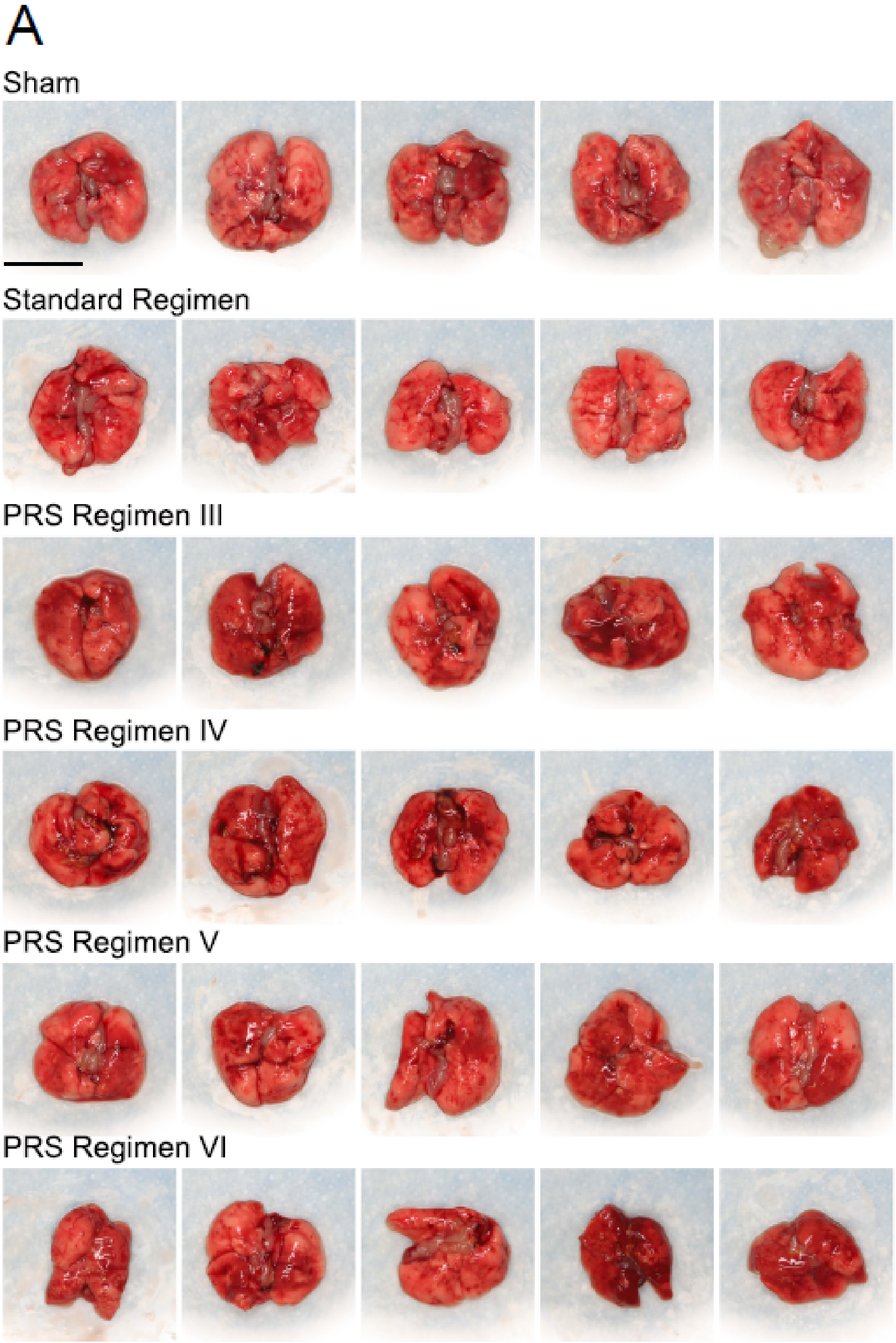
A
Sham
Standard Regimen
PRS Regimen III
PRS Regimen IV
PRS Regimen V
PRS Regimen VI

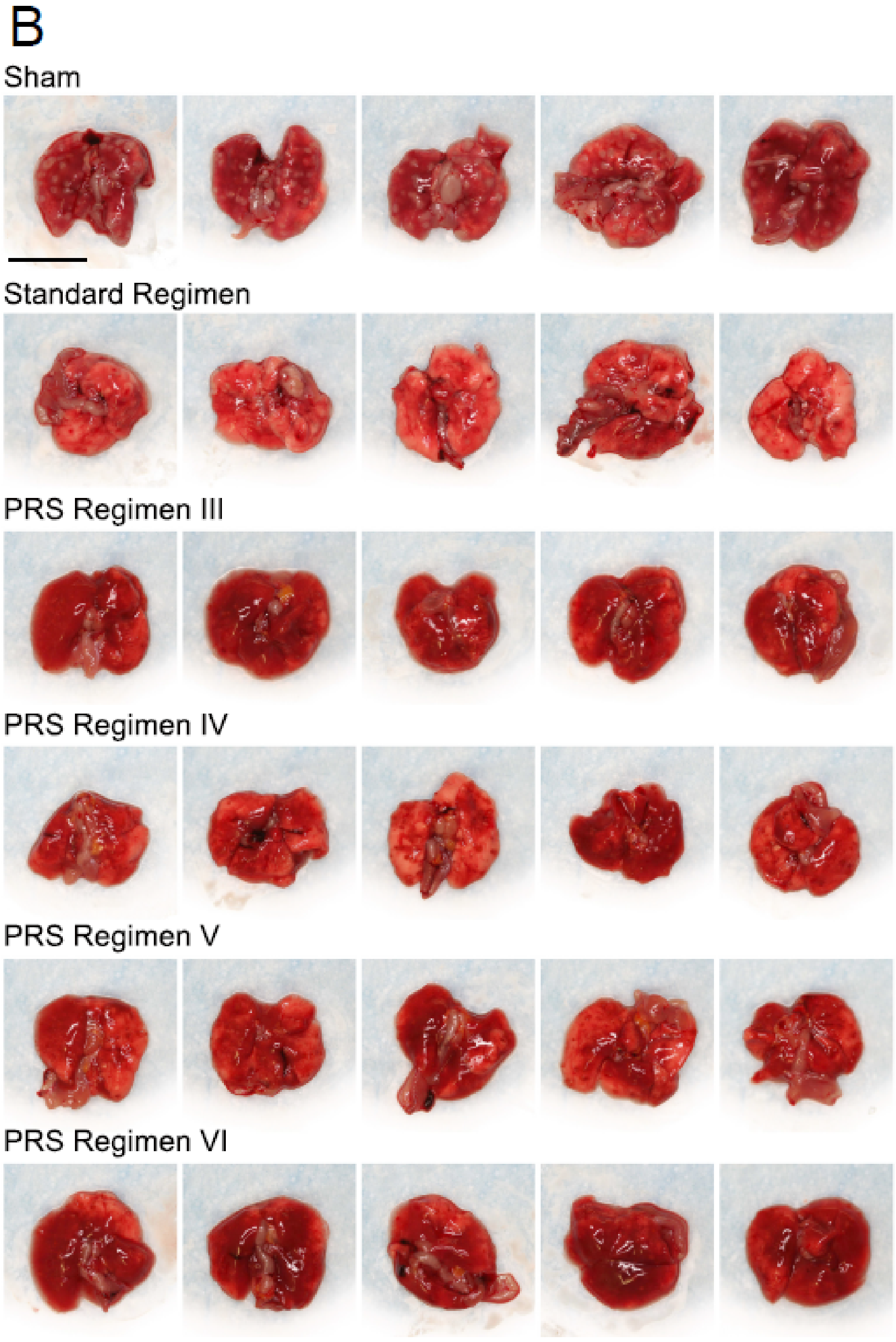
B
Sham
Standard Regimen
PRS Regimen III
PRS Regimen IV
PRS Regimen V
PRS Regimen VI

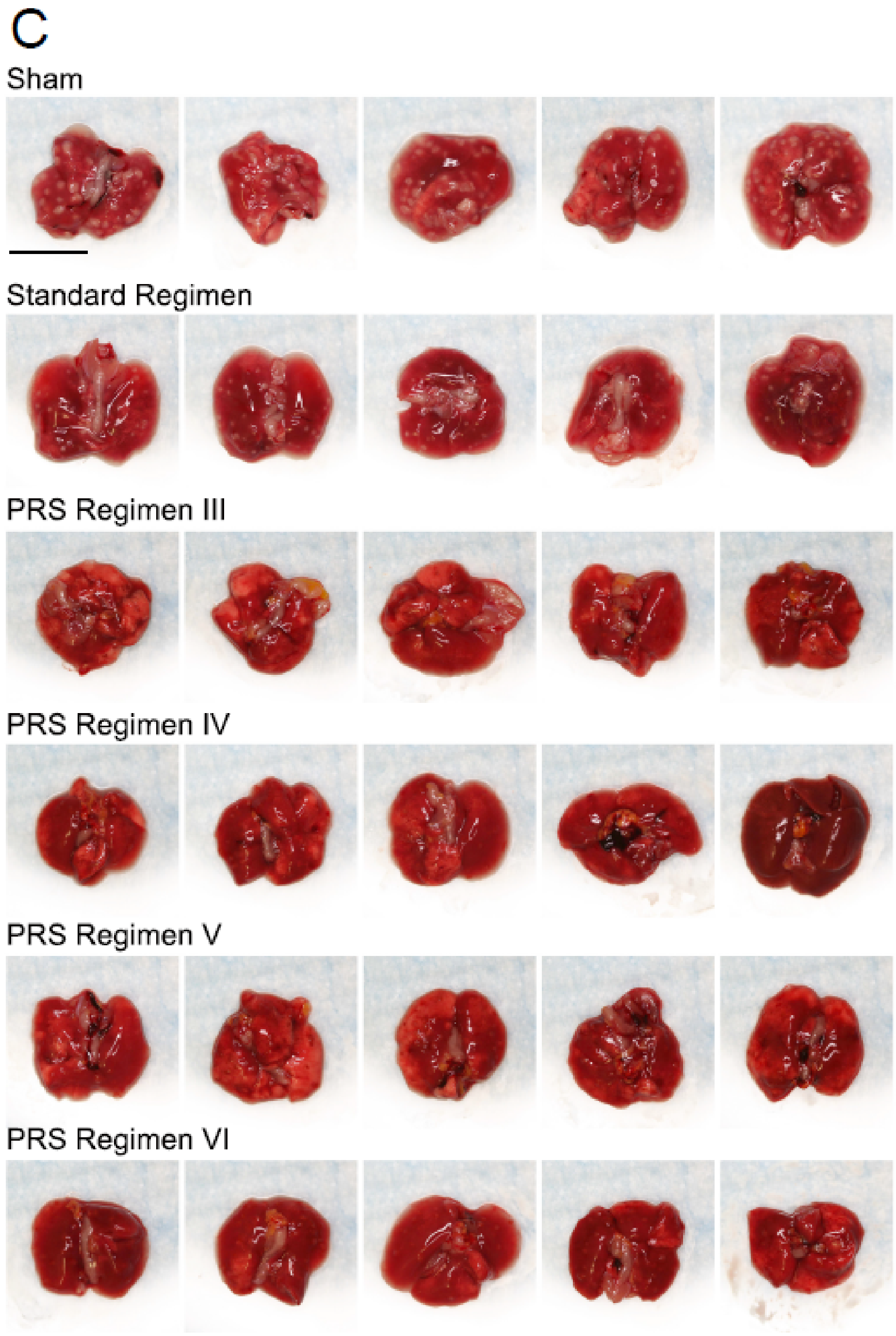

S2 Fig. Lung pathology in mice upon completion of 3, 4, and 5 weeks of treatment. Mice were infected with *M. tuberculosis* Erdman by aerosol and two weeks later received sham treatment or treatment with the Standard Regimen (INH, RIF, EMB, PZA at 25, 10, 100, 150 mg/kg), PRS Regimen III (CFZ, BDQ, PZA, SQ109 at 25, 30, 450, 25 mg/kg), PRS Regimen IV (CFZ, BDQ, PZA, AC at 25, 37, 50, 66.7-16.7 mg/kg), PRS Regimen V (CFZ, BDQ, PZA, DLM at 25, 40, 185, 0.83 mg/kg) or PRS Regimen VI (CFZ, BDQ, PZA at 25, 40, 185 mg/kg) 5 days per week for (A) 3 weeks, (B) 4 weeks, and (C) 5 weeks. The mice were euthanized upon treatment completion and their lungs were imaged. Scale bar (upper left panel), 1 cm.
